# Cytotoxic Indolocarbazoles From a Marine-Derived *Streptomyces* Sp. OUCMDZ-5380

**DOI:** 10.3389/fmicb.2022.957473

**Published:** 2022-07-12

**Authors:** Tongxu Cui, Simin Lin, Zizhen Wang, Peng Fu, Cong Wang, Weiming Zhu

**Affiliations:** ^1^Key Laboratory of Marine Drugs, Ministry of Education of China, School of Medicine and Pharmacy, Ocean University of China, Qingdao, China; ^2^Laboratory for Marine Drugs and Bioproducts, Pilot National Laboratory for Marine Science and Technology, Qingdao, China; ^3^Key Laboratory of Chemistry and Engineering of Forest Products, State Ethnic Affairs Commission, School of Chemistry and Chemical Engineering, Guangxi Minzu University, Nanning, China

**Keywords:** indolocarbazoles, streptocarbazoles F–H, antiproliferatory activity, marine microorganism, *Streptomyces* sp.

## Abstract

Under the guidance of global natural product social molecular networking, three new indolocarbazoles named streptocarbazoles F–H (**1**-**3**), along with staurosporine (**4**) were isolated from the marine-derived *Streptomyces* sp. OUCMDZ-5380. Structures of streptocarbazoles F–H were, respectively, determined as *N*-demethyl-*N*-hexanoylstaurosporine (**1**), *N*-demethyl-*N*-(2-methyl-3-methoxypyridin-4-yl) staurosporine staurosporine (**2**), and 4-(*N*-demethylstaurosporine-*N*-yl)-1,2-dimethyl-3-methoxypyridinium (**3**) by spectroscopic analysis and electronic circular dichroism comparison with staurosporine. Compared with staurosporine (**4**), streptocarbazoles F–H (**1**-**3**) showed a selective antiproliferation of the acute myeloid leukemia cell line MV4-11 with the IC_50_ values of 0.81, 0.55, and 1.88 μM, respectively.

## GRAPHICAL ABSTRACT

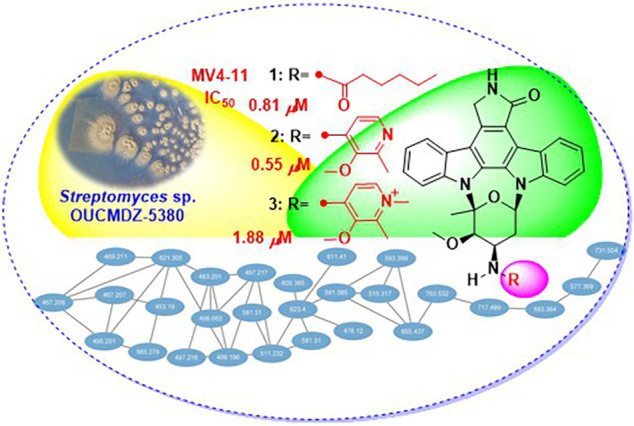

## Introduction

Staurosporine and its analogs are a special family of natural products with an indolo[2,3-*a*]pyrrolo [3,4-*c*]carbazole-12,13-diyl *N*-2-deoxy-1,5-glucopyranoside framework, which have attracted great attention because of the interesting structures and diverse bioactivities (Bergman et al., 2001; Sánchez et al., 2006; Janosik et al., 2008, 2018; Zenkov et al., 2020; Chambers et al., 2021). Since the first indolocarbazole, staurosporine was reported in 1977 (Ômura et al., 1977), more than 55 staurosporine derivatives have been isolated and identified from different organisms, namely, bacteria, clams, ascidia, slugs, and tunicates (Zenkov et al., 2020). Moreover, midostaurin (Stone et al., 2018), a staurosporine derivative, had been developed as the antitumor drug for the treatment of acute myeloid leukemia (AML). In recent years, the discovery of new compounds has been hampered due to the repeated isolation of known compounds. Therefore, dereplication becomes critical to finding new bioactive molecules. The LC-MS/MS-based global natural product social (GNPS) molecular networking can provide guidance and improve efficiency in the discovery of new and bioactive natural products within complex mixtures by analyzing and visualizing the spectral similarity of MS/MS data (Guthals et al., 2012; Watrous et al., 2012; Quinn et al., 2017). Molecular networking plays a significant role in exploring correlations of chemical structures among the known compounds, their analogs, and new compounds, which can guide the separation of the unreported compounds (Cheng et al., 2018).

We previously identified a staurosporine-producing strain, *Streptomyces* sp. OUCMDZ-5380 (originally numbered as MDCW-126), from a marine driftwood sample (Wang et al., 2019). To discover new indolocarbazoles cytotoxic to MV4-11, a human AML cell line with internal tandem duplication (ITD) mutation, we carried out the optimization of fermentation conditions for *Streptomyces* sp. OUCMDZ-5380 in different media. The ethyl acetate (EtOAc) or acetone extracts of the fermentation broth in different media were first analyzed by HPLC-UV at λ_max_ 290 nm (Supplementary Figure 1) to evaluate the chemodiversity of staurosporine analogs and assayed the cytotoxic activity against MV4-11 cells. Kazuo medium was selected as the optimal medium for the EtOAc extract containing more variety of staurosporine analogs and showing the most effective inhibition (99.2%) against the proliferation of MV-4-11 cells at 0.1 mg/ml (Supplementary Figure 1).

**FIGURE 1:**
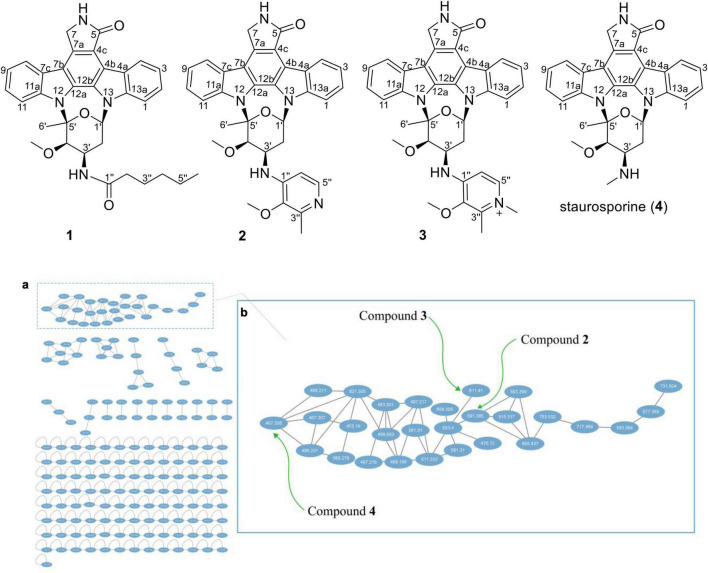
Structures **1**–**4** and the molecular networking of the extract (MeOH layer) from *Streptomyces* sp. OUCMDZ-5380 (a) and the compound cluster of indolocarbazole family observed in the molecular networking (b).

Thus, 113 L scale fermentation of *Streptomyces* sp. OUCMDZ-5380 in Kazuo medium was subsequently performed and the fermentation broth was extracted with acetone and EtOAc to give an organic extract. The organic extract was subjected to LC-MS/MS analysis which was then converted and generated a visualized GNPS molecular networking (Figure 1; Quinn et al., 2017). Compound **4** with *m/z* 467.20 [M+H]^+^ (Supplementary Figure 2) could be proposed as staurosporine (C_28_H_27_N_4_O_3_), which was confirmed by the follow-up isolation and identification (Supplementary Table 1 and Supplementary Figures 3, 4). Two of the 28 nodes visualized in the cluster of indolocarbazole family, that is, *m/z* 591.3 [M+NH_4_]^+^ (Supplementary Figure 12) for **2** and *m/z* 611.4 [M+Na]^+^ (Supplementary Figure 20) for **3**, could be new indolocarbazoles. Chemical isolation guided by molecular networking and LC-MS provided compounds **1**-**4** from the fermentation extract. Compounds **1**-**3** were identified as new staurosporine derivatives that we named streptocarbazoles F–H, while compound **4** was the known staurosporine (Fu et al., 2012).

## Materials and Methods

### General Experimental Procedures

Ultraviolet (UV) spectra were recorded on a Thermofisher NanoDrop OneC microvolume UV-Vis spectrophotometer. Experimental electronic circular dichroism (ECD) spectra in MeOH were recorded on a Chirascan circular dichroism spectrometer. IR spectra were obtained on a Nicolet Nexus 470 spectrophotometer using KBr disks. NMR spectroscopic data were recorded on Bruker Avance NEO 400 MHz, Agilent DD2-500, and JEOL JNM-ECP600 spectrometers, where chemical shifts were referenced to the corresponding residual solvent signal (δ_H/C_ 2.50/39.52 for DMSO-*d*_6_). High-resolution ESI-TOF mass spectra were recorded using a Waters Q-TOF Ultima Global GAA076 LC mass spectrometer. LC-MS/MS was performed on an Agilent Infinity 1290 HPLC instrument, coupled with a Thermo Electron LTQ-Orbitrap XL mass spectrometer, with a C_18_ column (YMC-Triart C_18_ column, 150 mm × 4.6 mm, 3-5 μm). Low-resolution LC/ESI-MS data were measured using a Waters ACQUITY SQD 2 UPLC/MS system with a reversed-phase C_18_ column (ACQUITY UPLC BEH C_18_, 2.1 mm × 50 mm, 1.7 μm) at a flow rate of 0.4 ml/min. Semipreparative HPLC was performed using an ODS column (YMC-park ODS-A, 10 mm × 250 mm, 5 μm). TLC was performed on plates precoated with silica gel GF254 (10-40 μm, Marine Chemical Factory of Qingdao). Silica gel (200-300 or 100-200 mesh, Marine Chemical Factory of Qingdao) was used for vacuum-liquid chromatography (VLC). RP-18 silica gel (YMC ODS-A, 50 μm) and Toyopearl HW-40F (Tosoh Bioscience) were used for column chromatography. Natural seawater was collected from the Huanghai Sea in the Jiaozhou Bay (Qingdao, China) without treatment.

### Strain and Fermentation Optimization Experiments

*Streptomyces* sp. OUCMDZ-5380 (originally numbered as MDCW-126) was isolated from a driftwood sample collected at the Beibu Gulf nearby Guangxi (21°44′39″N and 108°35′22″E) and identified by the 16S rRNA gene sequence (GenBank accession no. MK615749; Wang et al., 2019). *Streptomyces* sp. OUCMDZ-5380 was seeded in 500 ml Erlenmeyer flasks each containing 150 ml of ISP2 medium (pH 7.3) prepared by dissolving malt extract (10 *g*), yeast extract (2 *g*), and D-glucose (4 *g*) in 1 L of natural seawater and shaken for 3 days at 28^°^C and 180 rpm, which was used as the seed culture. Then the seed liquid (5 ml) was transferred to a 500 ml Erlenmeyer flask containing 150 ml of Kazuo medium (pH 7.5) that was prepared by dissolving soluble starch (25 *g*), yeast extract (2 *g*), soybean meal (15 *g*), CaCO_3_ (2 *g*), and Amberlite XAD-16 N (10 *g*) in 1 L natural seawater and was shaken on a rotary shaker at 180 rpm and 28^°^C for 9 days. The seed liquid (5 ml) was also aseptically transferred to rice solid medium and corn solid medium which were, respectively, prepared by adding rice (100 *g*) and corn (100 *g*) in 80 ml ISP2 medium, both of which were statically cultured at 28^°^C for 2 months. The fermentation broth in the Kazuo medium was filtrated into supernate and residue-containing resin and mycelia. The supernate was extracted three times with three volumes of EtOAc to give the EtOAc extract, and the residue was extracted three times with three volumes of acetone to give the acetone extract. The EtOAc and acetone extracts were combined and concentrated in a vacuum to give extract A. While fermentation on rice and corn solid media were extracted three times with three volumes of EtOAc to give extracts B and C, respectively.

### Scale Fermentation and Extraction

*Streptomyces* sp. OUCMDZ-5380 was seeded in an ISP2 medium and shaken for 3 days at 28^°^C and 180 rpm, which was used as the seed culture. Then the seed liquid (5 ml) was transferred to 500 ml Erlenmeyer flasks each containing 150 ml of Kazuo medium and shaken for 9 days at 28^°^C and 180 rpm. A total of 113.25 L cultivation was performed and extracted with acetone and EtOAc. By the same separation procedure, the fermentation liquor was extracted with EtOAc and acetone. The EtOAc and acetone extracts were combined and concentrated in a vacuum to give 242.12 *g* extract, which was partitioned between 90% MeOH/H_2_O and petroleum ether (PE) to give the MeOH/H_2_O layer (46.46 *g*).

### Molecular Networking Analysis and Its Application

The MeOH/H_2_O layer was dissolved in MeCN to prepare a 5 mg/ml solution and analyzed by LC-MS/MS. The sample was injected and eluted with a gradient program of MeCN-H_2_O containing 0.1% formic acid (0-20 min 10–100%; 0.5 ml/min; MS scan 200-2,000 Da). Mass spectra were obtained in positive ESI mode and with an automated full-dependent MS/MS scan. The MS/MS data were converted digitally to.mzXML file that was recognized by the GNPS using CompassXport software. The molecular networking was performed using the GNPS data analysis workflow. The spectral networks were imported into Cytoscape (version 3.9.1) and visualized using the force-directed layout.

As shown in Figure 1, each ellipse represented a compound with an *m/z* value. The connection lines indicated that the MS/MS fragments of the compounds were correlated, which could be used to identify the structural similarity of the compounds. From the complete molecular network, it could be seen that there were several compound clusters, and the one with the most nodes contained the reported staurosporine (Fu et al., 2012; *m/z* 467.2 [M+H]^+^). This compound cluster was thus useful for recognition of the indolocarbazole family, which contained new LC-MS peaks at *m/z* 591.3 and 611.4 corresponding to streptocarbazoles G (**2**; *m/z* 591.3 [M+NH_4_]^+^) and H (**3**; *m/z* 611.4 [M+Na]^+^), respectively. Similarly, the LC-MS/MS-based GNPS molecular networking could be used as guidance for isolating the new compounds from the extracts.

### Isolation and Purification

The MeOH layer (46.5 *g*) was subjected to a silica gel VLC column using step gradient elution with CH_2_Cl_2_-PE (0–100%) and then with MeOH-CH_2_Cl_2_ (0–100%) to yield eight fractions (Fr.1–Fr.8). Fr.3 (9.0 *g*) was re-chromatographed on a silica gel VLC column with a step gradient of MeOH-CH_2_Cl_2_ (0–100%) to provide nine fractions (Fr.3.1–Fr.3.9). Fr.3.5 (182.0 mg) was fractionated by Toyopearl HW-40F column eluting with MeOH-CH_2_Cl_2_ (1:1) to give ten fractions (Fr.3.5.1–Fr.3.5.10). Fr.3.5.1 (9.8 mg) was purified by semipreparative HPLC (ODS column, 44% MeCN-H_2_O with 0.05% TFA, 3 ml/min) to yield compound **1** (2.0 mg, *t*_*R*_ 77.0 min). Fr.8 (669.2 mg) was fractionated by Toyopearl HW-40F column eluting with MeOH-CH_2_Cl_2_ (1:1), to give three fractions (Fr.8.1-Fr.8.3). Fr.8.3 (342.8 mg) was subjected to an RP-silica gel column eluting with a step gradient of MeOH-H_2_O (20–100%) to afford four fractions (Fr.8.3.1–Fr.8.3.4). Fr.8.3.2 (15.6 mg) was purified by semipreparative HPLC [ODS column, 30% MeCN-H_2_O containing 0.05% trifluoroacetic acid (TFA), 4 ml/min] to yield compound **2** (3.0 mg, *t*_*R*_ 37.3 min). Fr.8.3.3 (18.3 mg) was purified by semipreparative HPLC (ODS column, 35% MeCN-H_2_O containing 0.05% TFA, 4 ml/min) to yield compound **3** (3.0 mg, *t*_*R*_ 35.5 min). Fr.4 (15.85 *g*) was purified by refluxing with acetone to afford compound **4** (8.0 *g*).

Streptocarbazole F (**1**): pale yellow powder; ${\left[\alpha \right]}_{\text{D}}^{19}$ +42.5 (*c* 0.05, MeOH); UV (MeOH) λ_max_ (log ε) 372 (3.43), 354 (3.39), 334 (3.59), 318 (3.60), 291 (4.17), and 238 (3.88) nm; IR (KBr) ν_max_ 3,433, 2,953, 2,359, 1,680, 1,520, 1,458, and 1,316 cm^–1^; ECD (0.45 mM, MeOH) λ_max_ 206 (Δε –9.57), 234 (Δε +3.73), 245 (Δε +1.41), 250 (Δε +1.83), 264 (Δε –0.28), and 295 (Δε +3.53) nm; ^1^H and ^13^C NMR, Table 1; and HRESIMS *m/z* 551.2651 [M+H]^+^ (calculated for C_33_H_35_N_4_O_4_, 551.2653).

**TABLE 1:** ^1^H (500 MHz) and ^13^C NMR (125 MHz) data for compounds **1**–**3** in DMSO-*d*_6_.

No.	1		2		3^a^	
	δ_C_, type	δ_H_, mult. (*J* in Hz)	δ_C_, type	δ_H_, mult. (*J* in Hz)	δ_C_, type	δ_H_, mult. (*J* in Hz)
1	108.9, CH	7.59, *d* (8.2)	109.1, CH	7.64, *d* (8.3)	109.1, CH	7.64, *d* (8.4)
2	125.3, CH	7.48, overlapped	125.5, CH	7.49, overlapped	125.2, CH	7.48, overlapped
3	119.4, CH	7.29, *t* (7.5)	119.7, CH	7.29, *t* (7.4)	119.8, CH	7.30, *t* (7.3)
4	125.7, CH	9.28, *d* (7.9)	125.7, CH	9.24, *d* (7.9)	125.7, CH	9.24, *d* (8.1)
4a	122.6, C	–	122.6, C	–	122.6, C	–
4b	114.9, C	–	115.1, C	–	115.1, C	–
4c	119.6, C	–	119.9, C	–	119.9, C	–
5	171.8, C	–	171.7, C	–	171.7, C	–
6	–	8.63, s	–	8.68, s	–	8.67, s
7	45.4, CH_2_	5.01, *d* (17.8); 4.96, d (17.8)	45.4, CH_2_	5.04, *d* (17.8); 4.99, d (17.8)	45.4, CH_2_	5.04, *d* (17.9); 4.99, d (17.9)
7a	132.3, C	–	132.6, C	–	132.6, C	–
7b	114.1, C	–	114.9, C	–	115.0, C	–
7c	123.9, C	–	124.3, C	–	124.3, C	–
8	121.3, CH	8.05, *d* (8.3)	121.2, CH	8.08, *d* (8.3)	121.3, CH	8.08, *d* (8.3)
9	120.4, CH	7.36, *t* (7.4)	120.8, CH	7.38, *t* (7.5)	120.9, CH	7.39, *t* (7.6)
10	125.1, CH	7.48, overlapped	125.2, CH	7.49, overlapped	125.5, CH	7.48, overlapped
11	114.3, CH	8.05, *d* (8.3)	115.6, CH	8.08, *d* (8.3)	115.6, CH	8.08, *d* (8.3)
11a	138.9, C	–	139.6, C	–	139.6, C	–
12a	128.8, C	–	125.3, C	–	125.0, C	–
12b	125.4, C	–	125.1, C	–	125.0, C	–
13a	136.3, C	–	136.7, C	–	136.7, C	–
1’	80.9, CH	6.87, *dd* (7.6, 3.3)	79.9, CH	6.92, *d* (6.2)	79.8, CH	6.92, *d* (6.2)
2’	29.5, CH_2_	2.41, *m*; 2.73, *m*	30.8, CH_2_	2.96, *m*; 2.71, *m*	30.7, CH_2_	2.95, *m*; 2.74, *d* (15.2)
3’	42.5, CH	4.43, *m*	44.5, CH	4.68, *m*	44.5, CH	4.67, brs
4’	81.2, CH	4.13, *d* (3.3)	79.7, CH	4.36, *d* (4.1)	79.6, CH	4.38, brs
4’-OCH_3_	59.1, CH_3_	2.87, s	58.1, CH_3_	3.23, s	58.0, CH_3_	3.26, s
5’	92.7, C	–	91.9, C	–	91.9, C	–
6’	29.0, CH_3_	2.39, s	28.8, CH_3_	2.36, s	28.8, CH_3_	2.36, s
3’-NH	–	6.77, *d* (6.8)	–	5.99, brs	–	5.98, *d* (7.2)
1”	171.9, C	–	150.9, C	–	149.9, C	–
2”	35.2, CH_2_	1.47, *m*; 1.61, *m*	139.6, C	–	140.2, C	–
2”-OCH_3_			59.1, CH_3_	2.43, s	59.4, CH_3_	2.36, s
3”	24.2, CH_2_	0.85, overlapped	143.0, C	–	145.5, C	
3”-CH_3_			13.9, CH_3_	2.10, s	12.7, CH_3_	2.11, s
4”	30.5, CH_2_	0.83, overlapped				
4”-CH_3_					42.8, CH_3_	3.68, s
5”	21.6, CH_2_	1.00, *d* (7.3); 0.98, d (7.0)	137.7, CH	8.01, *d* (6.9)	142.3, CH	8.14, *d* (7.4)
6”	13.7, CH_3_	0.69, *t* (7.3)	104.3, CH	7.03, *d* (6.9)	104.1, CH	7.08, *d* (7.4)

*^a^Recorded in 600 and 150 MHz for ^1^H and ^13^C NMR, respectively.*

Streptocarbazole G (**2**): pale yellow powder; ${\left[\alpha \right]}_{\text{D}}^{17}$ +6.6 (*c* 0.05, MeOH); UV (MeOH) λ_max_ (log ε) 371 (3.46), 354 (3.42), 334 (3.63), 319 (3.58), 290 (4.25), and 243 (3.92) nm; IR (KBr) ν_max_ 3,403, 2,932, 2,370, 1,684, 1,541, 1,458, 1,384, 1,210, 1,136, and 1,025 cm^–1^; ECD (0.44 mM, MeOH) λ_max_ 204 (Δε –6.67), 223 (Δε +4.00), 240 (Δε –3.42), 260 (Δε –0.31), 276 (Δε –2.37), and 295 (Δε +4.74) nm; ^1^H and ^13^C NMR, Table 1; and HRESIMS *m/z* 574.2440 [M + H]^+^ (calculated for C_34_H_32_N_5_O_4_, 574.2449).

Streptocarbazole H (**3**): brown oily liquid; ${\left[\alpha \right]}_{\text{D}}^{17}$ +18.5 (*c* 0.05, MeOH); UV (MeOH) λ_max_ (log ε) 371 (3.89), 354 (3.86), 333 (4.10), 318 (4.08), 290 (4.70), and 243 (4.32) nm; IR (KBr) ν_max_ 3,484, 2,927, 2,359, 1,682, 1,635, 1,557, 1,458, 1,384, 1,317, 1,201, 1,131, and 1,028 cm^–1^; ECD (0.43 mM, MeOH) λ_max_ 204 (Δε –8.89), 224 (Δε +5.86), 240 (Δε –4.64), 266 (Δε –0.28), 279 (Δε –3.04), and 297 (Δε +7.22) nm; ^1^H and ^13^C NMR, Table 1; and HRESIMS *m/z* 588.2607 M^+^ (calculated for C_35_H_34_N_5_O_4_^+^, 588.2605).

### Antiproliferatory Activity Assay

The antiproliferatory activity of compounds **1**–**3** was evaluated against 12 human cancer cell lines (lung adenocarcinoma cell line A549, lung adenocarcinoma cell line PC-9 with EGFR mutation, gastric cancer cell line MKN-45, colorectal carcinoma cell line HCT-116, acute promyelocytic leukemia cell line HL-60, erythroleukemic cell line K562, acute myeloid leukemia cell line MV4-11 with ITD mutation, acute human leukemia monocytic cell line THP-1 to constitutively express most TLRs, acute T lymphocyte cell line Jurkat, pancreatic cancer cell line PATU8988T, hepatocellular carcinoma cell line HepG2, and hepatoma cell line HuH-7) and one human cell line L-02 (embryo liver cell line) using the CCK-8 assay (Tominaga et al., 1999). These cells were, respectively, prepared into single-cell suspension with the RPMI1640 or MEDM medium containing 10% FBS, and the 96 well plates were inoculated with 100 μl cell culture medium (adherent cell viewed 5 × 10^4^/ml and suspension cell viewed 9 × 10^4^/ml) per well, then cultured at 5% CO_2_ and 37^°^C for 24 h before being exposed to each compound at eight concentration gradients. Then the 96 well plates adding the compounds were cultured for 48 h. The old culture medium and drug solution of adherent cells were sucked out, then 100 μl of CCK-8 solution (diluted ten times with the basic medium) was added and the suspension cells were directly added to 10 μl of CCK-8 stock solution. After incubation at 37^°^C with 5% CO_2_ for 4 h away from light, the cell culture measured the absorbance at 450 nm. The IC_50_ was calculated by the software GraphPad Prism 8 (version 8.0.2, from GraphPad Software Inc). Staurosporine (**4**) was used as the positive control. By the same procedure, the inhibitory rates of extracts A–C on MV4-11 cells were also tested at the final concentration of 0.1 mg/ml.

## Results and Discussion

**FIGURE 2:**
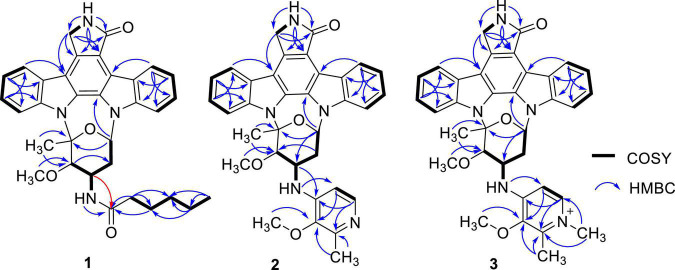
^1^H-^1^H COSY and HMBC correlations of compounds **1**–**3**.

**FIGURE 3:**
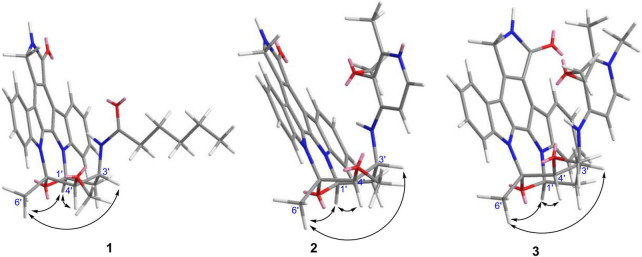
Key NOESY correlations of compounds **1**–**3**.

**FIGURE 4:**
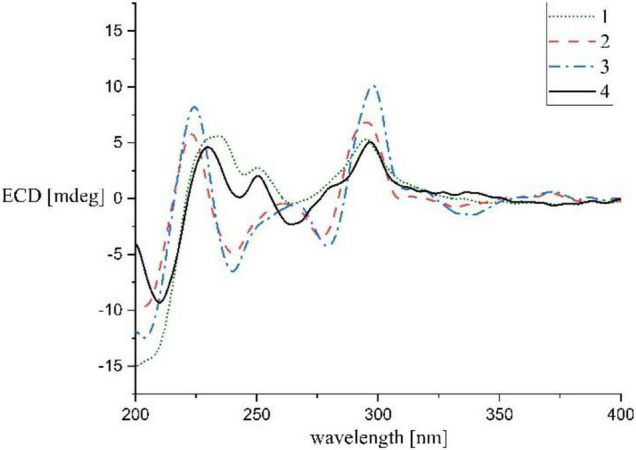
Experimental ECD spectra of compounds **1**–**4**.

Streptocarbazole F (**1**) was obtained as a pale-yellow powder. Its molecular formula was determined as C_33_H_35_N_4_O_4_ based on the HRESIMS data at *m/z* 551.2651 [M+H]^+^ (Supplementary Figure 5). Careful comparison of its ^1^H (Supplementary Figure 6) and ^13^C (Supplementary Figure 7) NMR data (Table 1) with those of staurosporine (**4**; Supplementary Table 1 and Supplementary Figures 3, 4) indicated an *N*-demethyl staurosporine unit that was confirmed by ^1^H-^1^H COSY (Supplementary Figure 9) of H-1/H-2/H-3/H-4, H-8/H-9/H-10/H-11, HN-6/H-7, H-1′/H-2′/H-3′/H-4′, and H-3′/HN-3′ and the HMBC (Supplementary Figure 10) correlations of H-1 to C-3 and C-4a, H-2 to C-4 and C-13a, H-3 to C-1 and C-4a, H-4 to C-2 and C-4b, HN-6 to C-4c and C-7a, H-7 to C-5 and C-7b, H-8 to C-11a and C-7b, H-9 to C-7c and C-11, H-10 to C-8 and C-11a, H-11 to C-9 and C-7c, H-1′ to C-12b and C-5′, H-2′ to C-4′, H-4′ to C-2′ and CH_3_O-4′, CH_3_O-4′ to C-4′, and H-6′ to C-4′ and C-5′ (Figure 2). In addition to the signals of the *N*-demethyl staurosporine unit, compound **1** also included the signals of a carbonyl (δ_C_ 171.9, C-1″), four sp^3^-methylene groups (δ_H/C_ 1.47 and 1.61/35.2, CH_2_-2″; 0.85/24.2, CH_2_-3″; 0.83/30.5, CH_2_-4″; and 0.98 and 1.00/21.6, CH_2_-5″), and a methyl group (δ_H/C_ 0.69/13.7, CH_3_-6″; Supplementary Figure 8). ^1^H-^1^H COSY (Supplementary Figure 9) of H-2″/H-3″/H-4″/H-5″/H-6″ and the HMBC (Supplementary Figure 10) correlations of H-2″ to C-1″, C-3″, and C-4″, and H-6″ to C-4″ and C-5″ (Figure 2) suggested that these groups connected as a hexanoyl moiety. The key HMBC (Supplementary Figure 10) correlations from H-3′ (δ_H_ 4.43) and HN-3′ (δ_H_ 6.77) to C-1″ attached the hexanoyl group to the N-3′ position. The relative configuration of **1** was elucidated by the NOESY (nuclear Overhauser effect spectroscopy) experiment which showed correlations of H-1′ (δ_H_ 6.87) to H-4′ (δ_H_ 4.13) and H-6′ (δ_H_ 2.39) and H-3′ (δ_H_ 4.43) to H-6′ (Figure 3 and Supplementary Figure 11), suggesting the same relative configuration to staurosporine (**4**). Moreover, compound **1** displayed similar ECD Cotton effects to staurosporine at λ_max_ 206 (Δε –9.57), 234 (Δε +3.73), and 295 (Δε +3.53) nm (Figure 4 and Supplementary Figure 28), indicating that compound **1** and staurosporine shared the same (1′*R*, 3′*R*, 4′*R*, and 5′*S*)—configuration (Funato et al., 1994; Fu et al., 2012). To further confirm the absolute configuration, the predicted ECD spectrum of compound **1** was obtained by the TDDFT [B3LYP/6-31G(d)] method (Stephens et al., 2001). The measured ECD spectrum of compound **1** matches with the calculated ECD curve of (1′*R*, 3′*R*, 4′*R*, and 5′*S*)-**1** (Supplementary Figure 28). Thus, streptocarbazole F (**1**) was identified as *N*-demethyl-*N*-hexanoyl staurosporine.

Streptocarbazole G (**2**) was obtained as a pale-yellow powder. Its molecular formula was determined as C_34_H_32_N_5_O_4_ based on the HRESIMS data at *m/z* 574.2440 [M+H]^+^ (Supplementary Figure 12). The comparison of the ^1^H (Supplementary Figure 13) and ^13^C NMR (Supplementary Figure 14) data of compound **2** (Table 1) with staurosporine (**4**; Supplementary Figures 3, 4) indicated the lack of 3′-*N*-methyl signals at δ_H/C_ 1.46/33.3 (Supplementary Table 1), showing an *N*-demethyl staurosporine unit in compound **2**. The rest signals included those for a methyl (δ_H/C_ 2.10/13.9, CH_3_-3″), a methoxy (δ_H/C_ 2.43/59.1, CH_3_O-2″), two sp^2^ methines (δ_H/C_ 8.01/137.7, CH-5″ and 7.03/104.3, CH-6″), and three sp^2^ nonhydrogen carbons (δ_C_ 150.9, C-1″; 139.6, C-2″; and 143.0, C-3″; Table 1; and Supplementary Figures 15, 16). Combined with the molecular formula, the ^1^H-^1^H COSY correlations of H-5″/H-6″ (Supplementary Figure 17) and the HMBC correlations (Supplementary Figure 18) from the olefinic protons H-5″ to C-1″ and C-3″, and H-6″ to C-2″ (Figure 2) presented a pyridine moiety. The HMBC (Supplementary Figure 18) correlations from the methoxy proton (δ_H_ 2.43) to C-2″ (δ_C_ 139.6) and the methyl proton (δ_H_ 2.10) to C-2″ and C-3″ (δ_C_ 143.0) indicated that the methoxy and methyl groups, respectively, linked to C-2″ and C-3″, forming a 2-methyl-3-methoxypyridine moiety. The key HMBC (Supplementary Figure 18) correlations of H-3′ (δ_H_ 4.68) to C-1″ (δ_C_ 150.9) and HN-3′ (δ_H_ 5.99) to C-6″ (δ_C_ 104.3) suggested that 2-methyl-3-methoxypyridine moiety and the *N*-demethylstaurosporine moiety were connected by a C–N σ-bond between N-3′ and C-1″ (Figure 2). The NOESY (Supplementary Figure 19) experiment showed correlations of H-1′ (δ_H_ 6.92) to H-4′ (δ_H_ 4.36) and H-6′ (δ_H_ 2.36) and H-3′ (δ_H_ 4.68) to H-6′ (Figure 3), suggesting the same relative configuration of **2** to staurosporine. Meantime, compound **2** displayed ECD Cotton effects at λ_max_ 204 (Δε –6.67), 223 (Δε +4.00), and 295 (Δε +4.74) nm (Figure 4) similar to those of staurosporine (Fu et al., 2012), indicating that compound **2** and staurosporine have the same absolute configuration. The 3D structure of compound **2** indicated that CH_3_O-2″ protons were shielded by the indolocarbazole framework (Figure 3), leading to an unusual chemical shift of 2″-methoxy protons (δ_H_ 2.43). Therefore, streptocarbazole G (**2**) was convincingly elucidated as *N*-demethyl-*N*-(2-methyl-3-methoxypyridin-4-yl) staurosporine.

Streptocarbazole H (**3**) was obtained as a brown oil. Its molecular formula was determined as C_35_H_34_N_5_O_4_^+^ based on the HRESIMS data at *m/z* 588.2607 [M]^+^ (Supplementary Figure 20), within a 15 amu more than **2**. Its ^1^H (Supplementary Figure 21) and ^13^C NMR (Supplementary Figure 22) data were very similar to those of **2** except for an additional methyl signal at δ_*C/H*_ 42.8/3.68 (Table 1 and Supplementary Figures 23–25). The HMBC (Supplementary Figure 26) correlations of this methyl proton signal (δ_H_ 3.68) to C-3″ (δ_C_ 145.5) and C-5″ (δ_C_ 142.3; Figure 3) located this methyl at the nitrogen atom of the pyridine ring, that is, 4″-position. Thus, compound **3** was deduced as the *N*-methyl pyridinium of compound **2** and was further evidenced by NOESY (Supplementary Figure 27) and ECD experiments. Compound **3** displayed the same NOE pattern from H-1′ (δ_H_ 6.92) to H-4′ (δ_H_ 4.38) and H-6′ (δ_H_ 2.36) and H-3′ (δ_H_ 4.67) to H-6′ (Figure 2), and the similar ECD Cotton effects to compound **2** (Figure 4), indicating that they share the same absolute configuration. Therefore, streptocarbazole H (**3**) was convincingly identified as the *N*-methyl pyridinium of **2**, that is 4-(*N*-demethyl staurosporine-*N*-yl)-1,2-dimethyl-3-methoxypyridinium.

Compounds **1**–**4** were assayed for their antiproliferative effects on twelve human cancer cell lines (A549, PC-9, MKN-45, HCT-116, HL-60, K562, MV4-11, THP-1, Jurkat, PATU8988T, HepG2, and HuH-7) and one human normal cell line (fetal hepatocyte line L-02) by CCK-8 method (Tominaga et al., 1999). The results showed that new compounds **1**-**3** exhibited a selective inhibitory activity against the growth of MV4-11 cancer cells with the IC_50_ values of 0.81 ± 0.007, 0.55 ± 0.04, and 1.88 ± 0.11 μM, respectively, while no activity against the other cell lines was not observed (IC_50_ ≥ 10 μM). The IC_50_ values for the positive control, staurosporine (**4**), were 0.12 ± 0.006, 13.58 ± 0.68, 0.0029 ± 0.0008, 0.062 ± 0.0006, 0.17 ± 0.018, 0.64 ± 0.073, 0.0014 ± 0.0001, 0.16 ± 0.038, >1, 0.12 ± 0.014, 0.18 ± 0.034, and 0.11 ± 0.016 μM against A549, PC-9, MKN-45, HCT-116, HL-60, K562, MV4-11, THP-1, Jurkat, PATU8988T, HepG2, HuH-7, and L-02 cell lines, respectively.

## Conclusion

In summary, three new indolocarbazoles, streptocarbazoles F–H (**1**-**3**), were identified from the marine-derived *Streptomyces* sp. OUCMDZ-5380 by spectroscopic analysis and ECD comparison. It is different from the broad-spectrum antiproliferative activity of staurosporine that streptocarbazoles F–H displayed a selective antiproliferation for the Flt3-ITD mutation AML cell line, MV4-11 with the IC_50_ values of 0.55–1.88 μM.

## Data Availability Statement

The original contributions presented in the study are included in this article/Supplementary Material, further inquiries can be directed to the corresponding authors.

## Author Contributions

All authors listed have made a substantial, direct, and intellectual contribution to the work, and approved it for publication.

## Conflict of Interest

The authors declare that the research was conducted in the absence of any commercial or financial relationships that could be construed as a potential conflict of interest.

## Publisher’s Note

All claims expressed in this article are solely those of the authors and do not necessarily represent those of their affiliated organizations, or those of the publisher, the editors and the reviewers. Any product that may be evaluated in this article, or claim that may be made by its manufacturer, is not guaranteed or endorsed by the publisher.
