# Supplementary material for: Cytotoxic Indolocarbazoles From a Marine-Derived Streptomyces Sp. OUCMDZ-5380
Source: Front Microbiol. 2022 Jul 12;13:957473. doi: 10.3389/fmicb.2022.957473 (PMC9315148; doi:10.3389/fmicb.2022.957473)
Supplement: Supplementary file 1 [file Data_Sheet_1.PDF]

Supporting Information

Cytotoxic Indolocarbazoles from a Marine-Derived *Streptomyces* sp. OUCMDZ-5380

Tongxu Cui[1#], Simin Lin [1#], Zizhen Wang[1], Peng Fu[1,2], Cong Wang[3*] and Weiming Zhu[1,2*]

[1]Key Laboratory of Marine Drugs, Ministry of Education of China, School of Medicine and Pharmacy, Ocean University of China, Qingdao 266003, China
[2]Laboratory for Marine Drugs and Bioproducts, Pilot National Laboratory for Marine Science and Technology (Qingdao), Qingdao 266237, China
[3]Key Laboratory of Chemistry and Engineering of Forest Products, State Ethnic Affairs Commission, School of Chemistry and Chemical Engineering, Guangxi Minzu University, Nanning 530006, China

List of Supporting Information

Figure. S1. HPLC-UV analysis and the inhibitory rate on MV4-11 cells at 0.1 mg/mL of the extracts from *Streptomyces* sp. OUCMDZ-5380……..S3

The Experimental Data of Compound 4……S3

Table S1. ^{1}H (400 MHz) and ^{13}C (100 MHz) NMR Data for Compound **4** in DMSO-d_6……S3

Figure S2. HRESIMS spectrum of staurosporine (**4**) ……S4

Figure S3. ^{1}H-NMR spectrum (400 MHz) of staurosporine (**4**) in DMSO-d_6……S5

Figure S4. ^{13}C-NMR spectrum (100 MHz) of staurosporine (**4**) in DMSO-d_6……S6

Figure S5. HRESIMS spectrum of streptocarbazole F (**1**)……S7

Figure S6. ^{1}H-NMR spectrum (500 MHz) of streptocarbazole F (**1**) in DMSO-d_6……S8

Figure S7. ^{13}C-NMR spectrum (125 MHz) of streptocarbazole F (**1**) in DMSO-d_6……S9

Figure S8. HSQC spectrum (500×125 MHz) of streptocarbazole F (**1**) in DMSO-d_6……S10

Figure S9. ^{1}H-^{1}H COSY spectrum (500×500 MHz) of streptocarbazole F (**1**) in DMSO-d_6……S11

Figure S10. HMBC spectrum (500×125 MHz) of streptocarbazole F (**1**) in DMSO-d_6……S12

Figure S11. NOESY spectrum (500×500 MHz) of streptocarbazole F (**1**) in DMSO-d_6……S13

Figure S12. HRESIMS spectrum of streptocarbazole G (**2**)……S14

Figure S13. ^{1}H-NMR spectrum (500 MHz) of streptocarbazole G (**2**) in DMSO-d_6……S15

Figure S14. ^{13}C-NMR spectrum (125 MHz) of streptocarbazole G (**2**) in DMSO-d_6……S16

Figure S15. DEPT 135-NMR (125 MHz) spectrum of streptocarbazole G (**2**) in DMSO-d_6……S17

Figure S16. HSQC spectrum (500×125 MHz) of streptocarbazole G (**2**) in DMSO-d_6……S18

Figure S17. ^{1}H-^{1}H COSY spectrum (500×500 MHz) of streptocarbazole G (**2**) in DMSO-d_6……S19

Figure S18. HMBC spectrum (500×125 MHz) of streptocarbazole G (**2**) in DMSO-d_6……S20

Figure S19. NOESY spectrum (500×500 MHz) of streptocarbazole G (**2**) in DMSO-d_6……S21

Figure S20. HRESIMS spectrum of streptocarbazole H (**3**)……S22

Figure S21. ^{1}H-NMR spectrum (600 MHz) of streptocarbazole H (**3**) in DMSO-d_6……S23

Figure S22. ^{13}C-NMR spectrum (150 MHz) of streptocarbazole H (**3**) in DMSO-d_6……S24

Figure S23. DEPT 135-NMR (150 MHz) spectrum of streptocarbazole H (**3**) in DMSO-d_6……S25

Figure S24. HSQC spectrum (600×150 MHz) of streptocarbazole H (**3**) in DMSO-d_6……S26

Figure S25. ^{1}H-^{1}H COSY spectrum (600×600 MHz) of streptocarbazole H (**3**) in DMSO-d_6……S27

Figure S26. HMBC spectrum (600×150 MHz) of streptocarbazole H (**3**) in DMSO-d_6……S28

Figure S27. NOESY spectrum (600×600 MHz) of streptocarbazole H (**3**) in DMSO-d_6……S29

Figure S28. Experimental and calculated ECD curves for **1** ……...S30

Table S2. DFT-optimized structures and thermodynamic parameters for low-energy conformers of **1**…....S31

Table S3. Optimized Z-matrixes of **1** in the gas phase (Å) at B3LYP/6-31G(d) level…....S33

Figure S1. HPLC-UV analysis and the inhibitory rate on MV4-11 cells at 0.1 mg/mL of the extracts from the fermentation broth of *Streptomyces* sp. OUCMDZ-5380

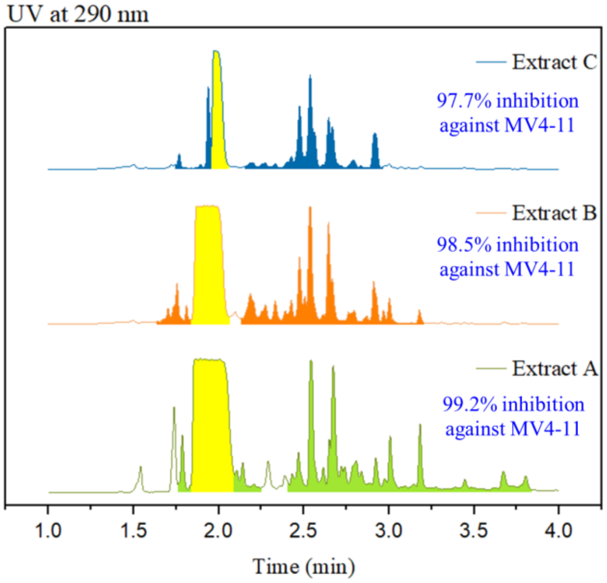

A was the EtOAc and acetone extract from Kazuo medium, B and C were the EtOAc extract respectively from rice solid medium and corn solid medium. Yellow peaks indicate staurosporine, blue, orange and green peaks indicate the staurosporine analogues.

The Experimental Data of Compound 4

Staurosporine (*4*): light yellow powder; $[\alpha]_D^{19.3}$ +49.1 (*c* 0.05, MeOH); UV (MeOH) λ_{max} (log ε) 373 (6.73), 356 (6.69), 343 (6.85), 335 (7.45), 292 (7.13), 241 (7.12) nm; ^{1}H and ^{13}C NMR, Table S1; HRESIMS *m/z* 467.2077 [M + H]$^+$ (calcd for $C_{28}H_{27}N_4O_3$, 467.2078).

Table S1. ^{1}H (400 MHz) and ^{13}C (100 MHz) NMR Data for Compound **4** in DMSO-d_6

**4**					
no.	δ_C, type	δ_H, mult. (*J* in Hz)	no.	δ_C, type	δ_H, mult. (*J* in Hz)
1	108.3, CH	7.58, d (8.2)	11	115.2, CH	7.99, overlapped
2	124.8, CH	7.45, overlapped	11a	139.4, C	-
3	119.0, CH	7.30, overlapped	12a	130.0, C	-
4	125.6, CH	9.33, d (7.9)	12b	126.7, C	-
4a	122.5, C	-	13a	136.3, C	-
4b	113.5, C	-	1'	79.9, CH	6.70, brs
4c	118.8, C	-	2'	29.4, CH_2	2.10, brs
5	172.3, C	-	3'	50.1, CH	3.25, m
6	-	8.55, s	3'-NCH_3	33.3, CH_3	1.46, s
7	45.4, CH_2	4.98, s	4'	82.8, CH	4.05, d (3.3)
7a	132.0, C	-	4'-OCH_3	57.2, CH_3	3.34, s
7b	114.1, C	-	5'	91.1, C	-
7c	123.9, C	-	6'	29.7, CH_3	2.31, s
8	120.8, CH	7.99, overlapped			
9	119.7, CH	7.30, overlapped			
10	124.3, CH	7.45, overlapped			

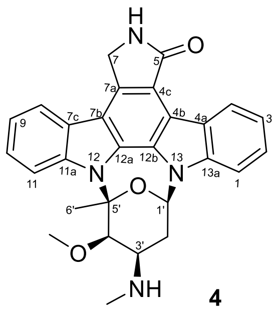

4

Figure S2. HRESIMS spectrum of staurosporine (**4**)

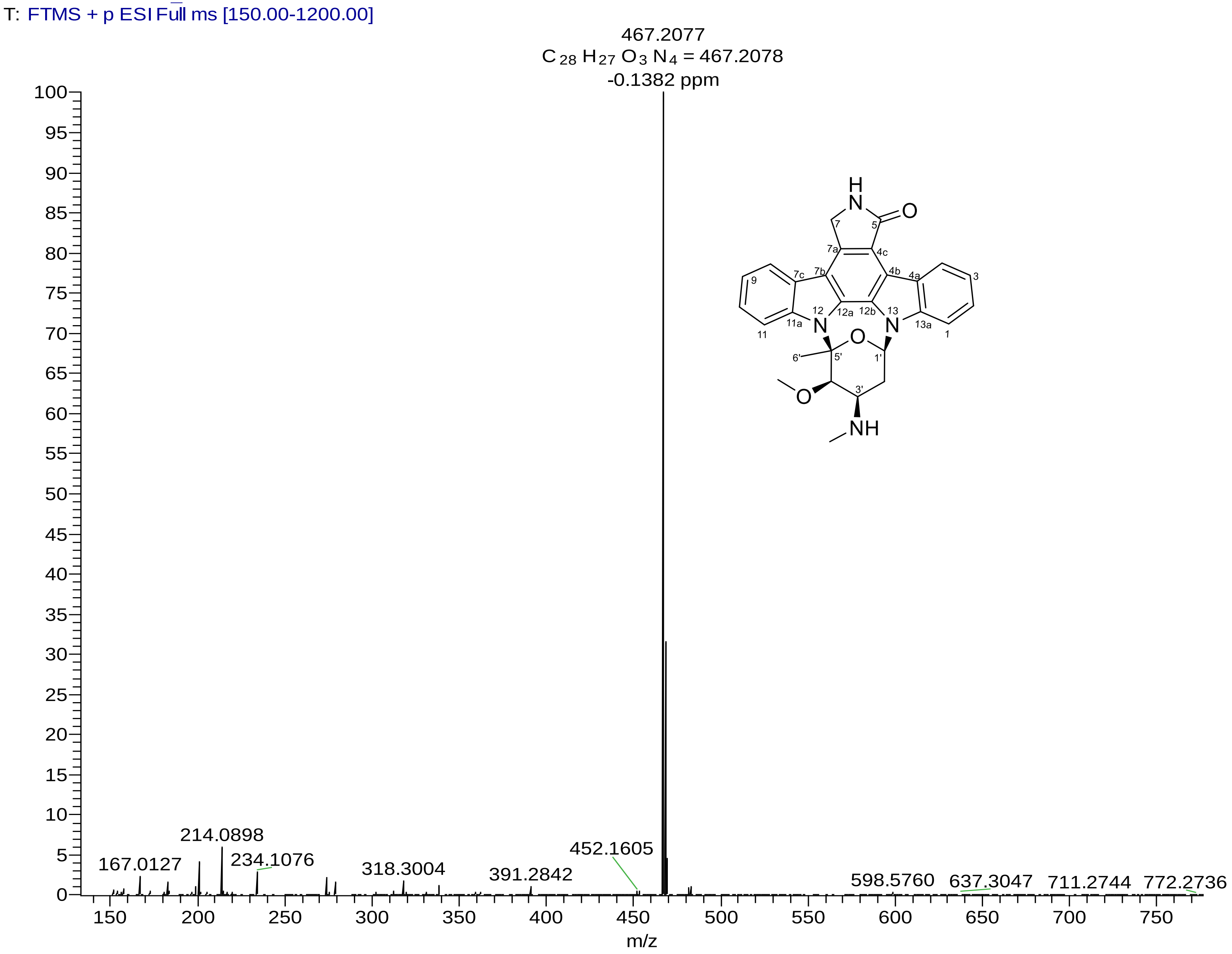

Figure S3. ^{1}H-NMR spectrum (400 MHz) of staurosporine (**4**) in DMSO-d_6

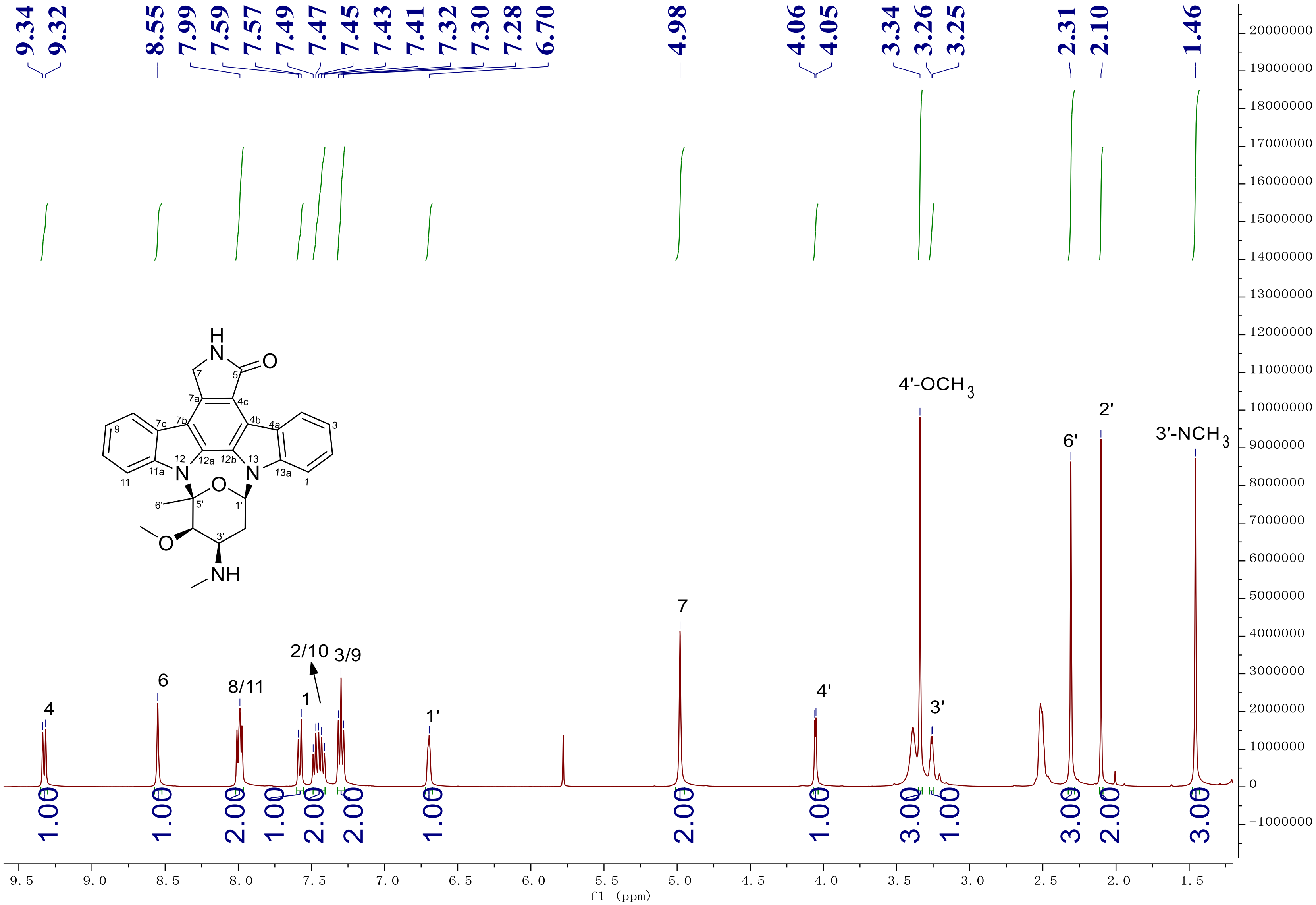

Figure S4. ^{13}C-NMR spectrum (100 MHz) of staurosporine (**4**) in DMSO-d_6

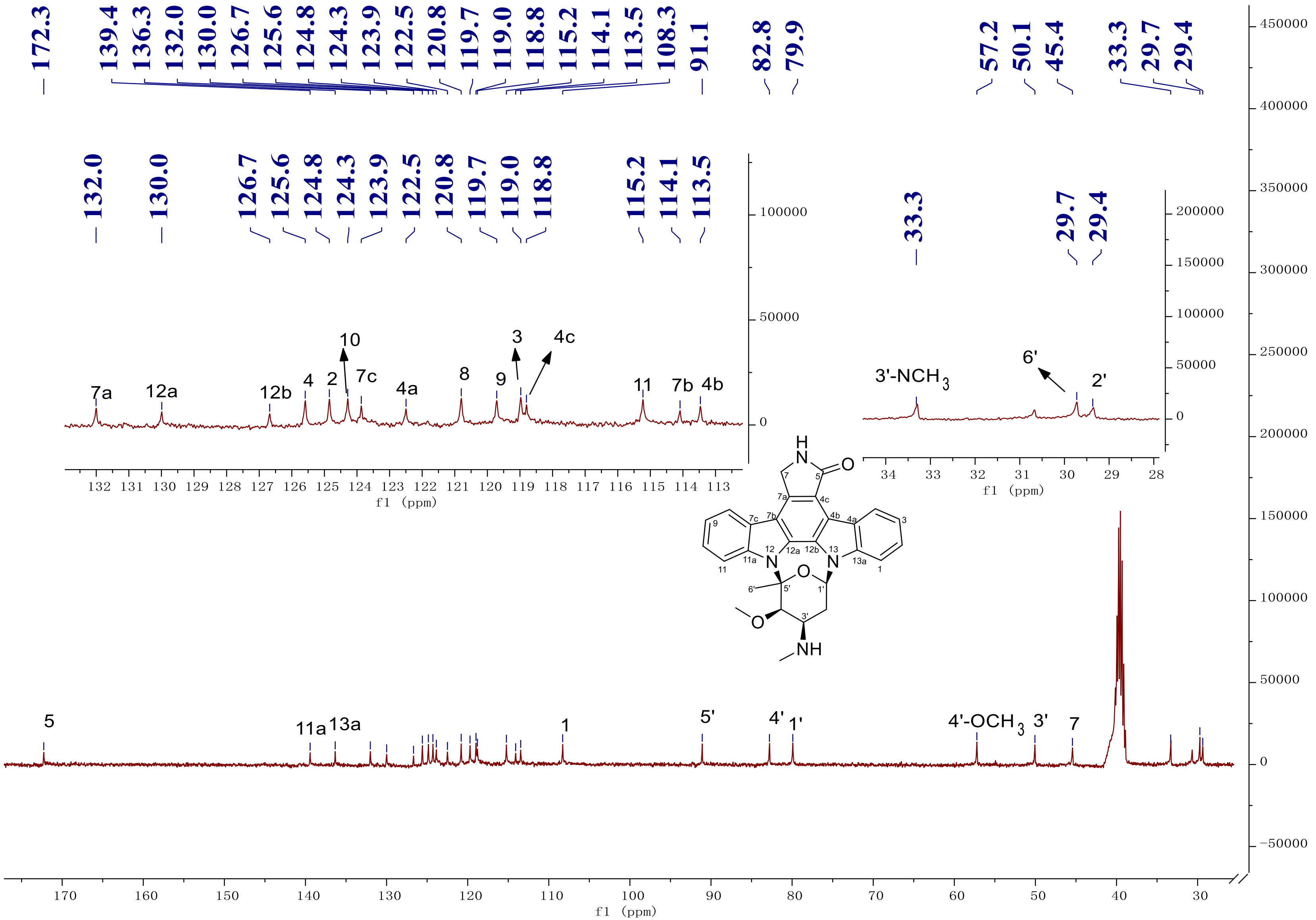

Figure S5. HRESIMS spectrum of streptocarbazole F (**1**)

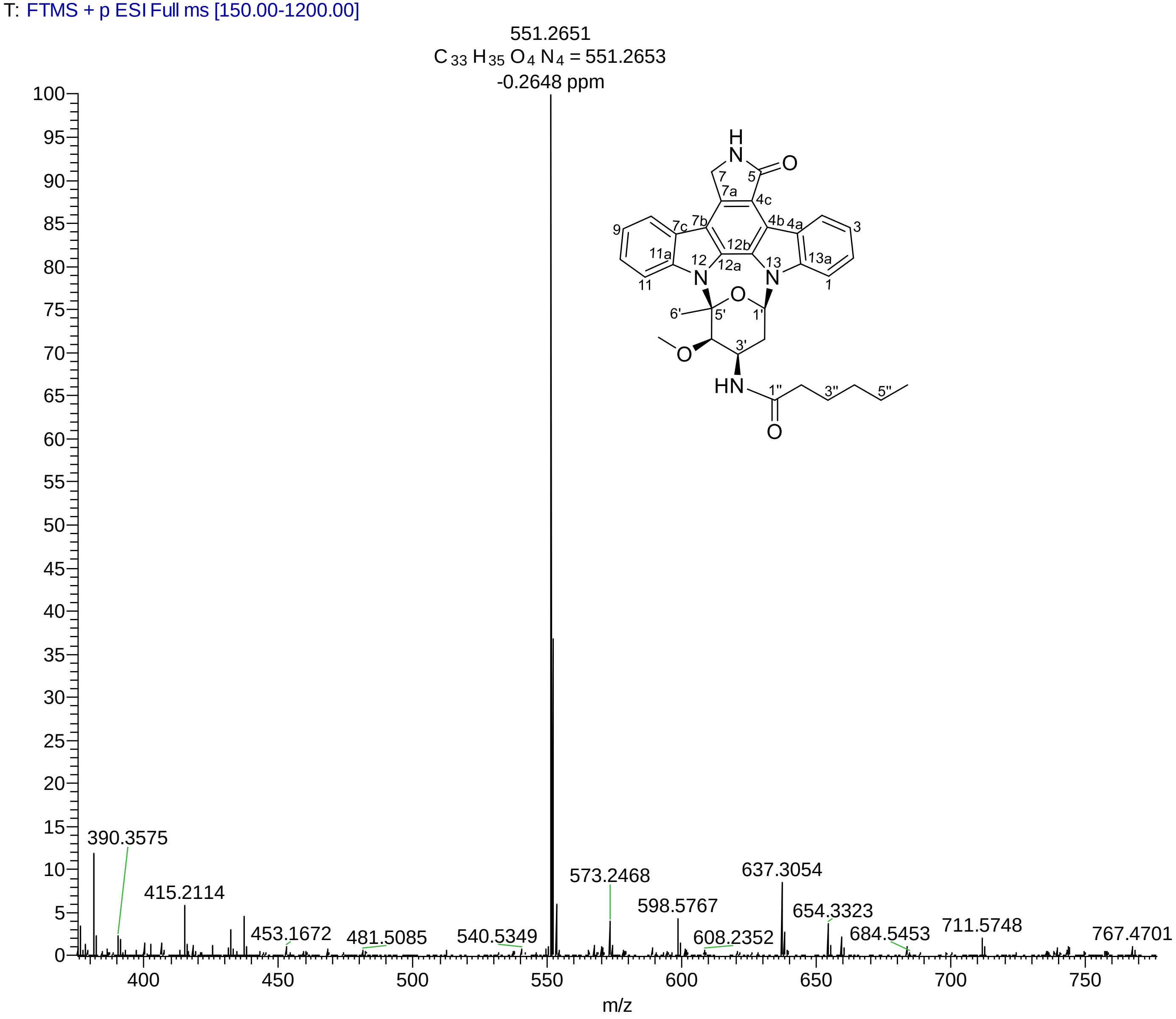

Figure S6. ^{1}H-NMR spectrum (500 MHz) of streptocarbazole F (**1**) in DMSO-d_6

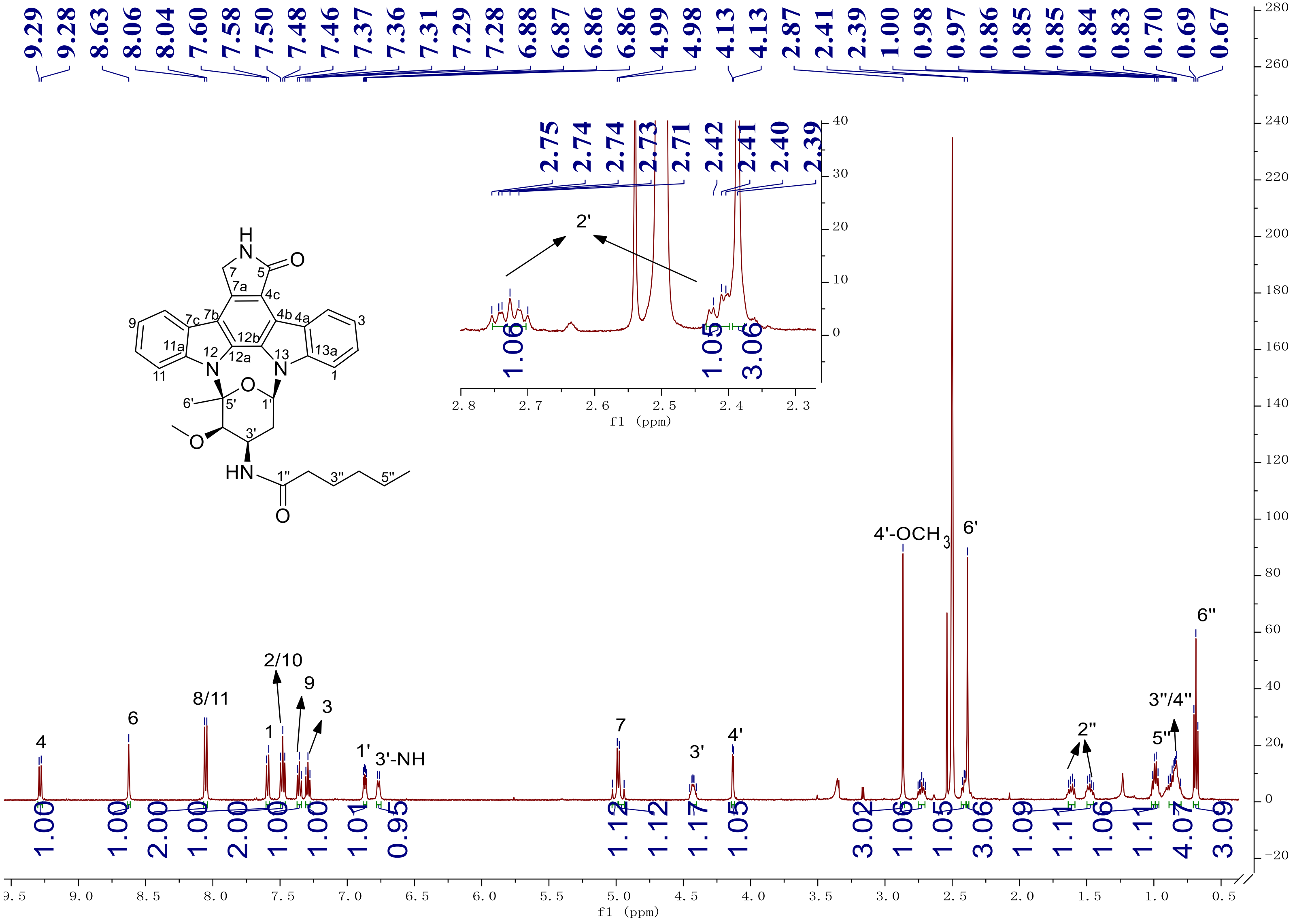

Figure S7. ^{13}C-NMR spectrum (125 MHz) of streptocarbazole F (**1**) in DMSO-d_6

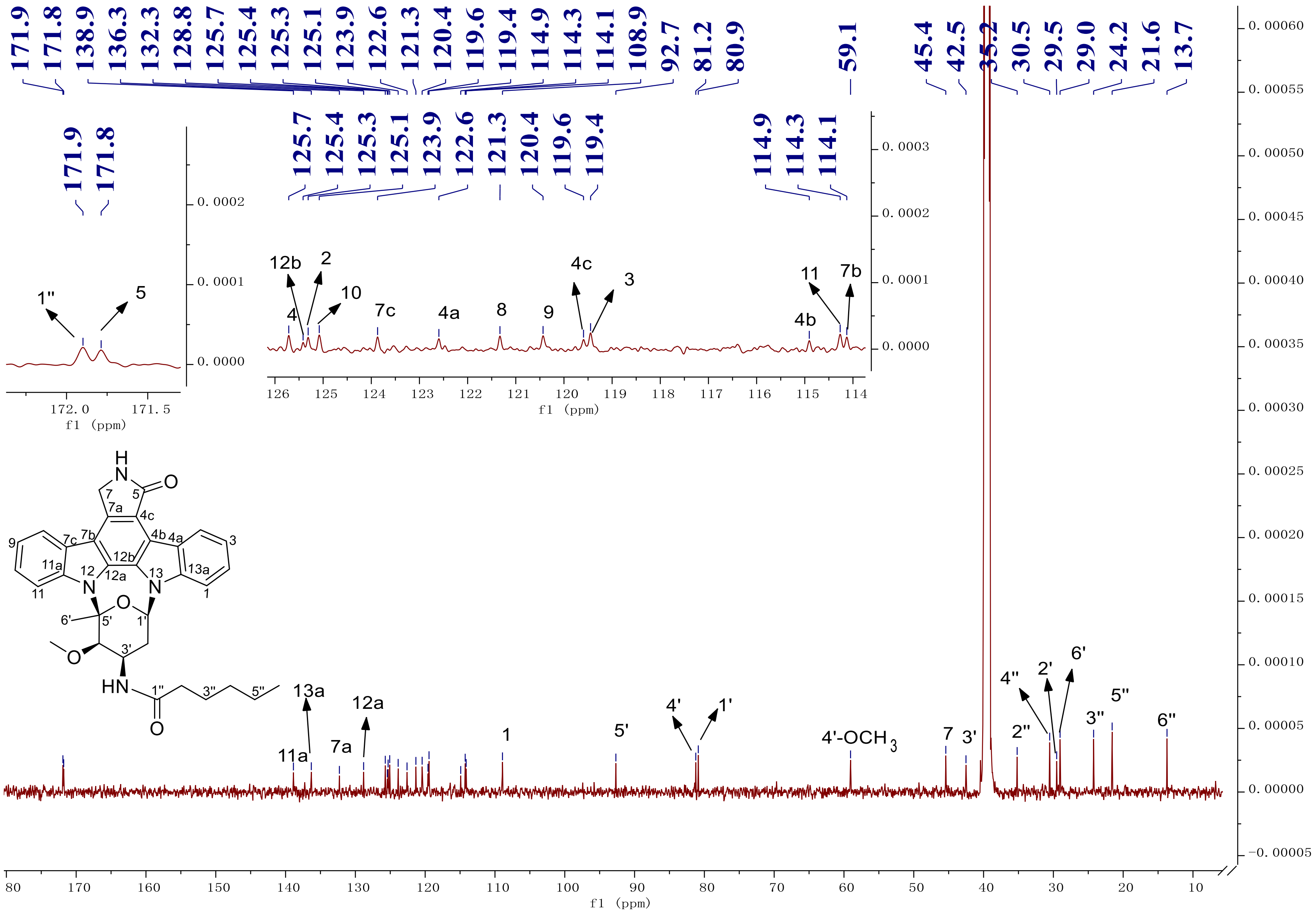

Figure S8. HSQC spectrum (500×125 MHz) of streptocarbazole F (**1**) in DMSO-d_6

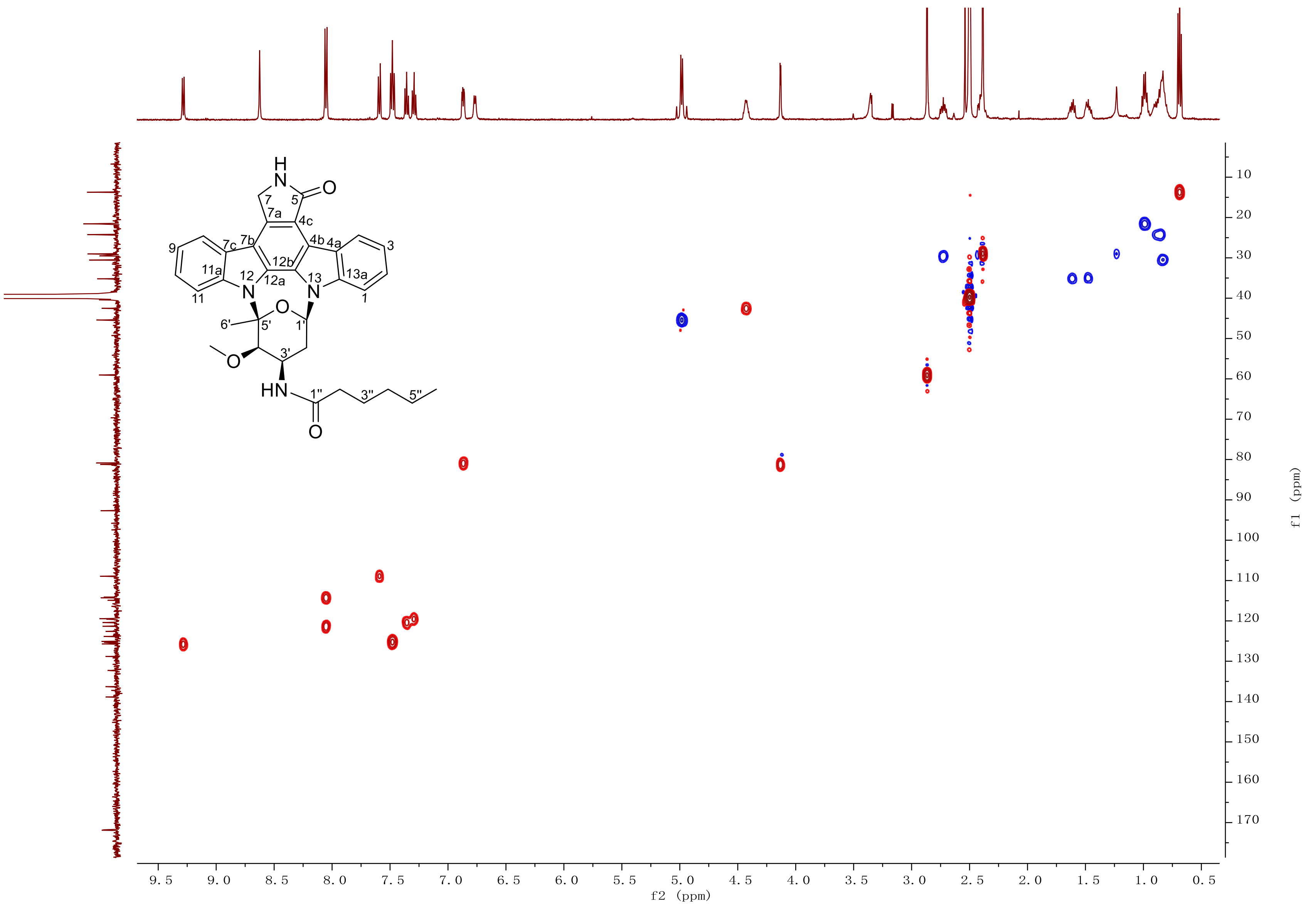

Figure S9. ^{1}H-^{1}H COSY spectrum (500×500 MHz) of streptocarbazole F (**1**) in DMSO-d_6

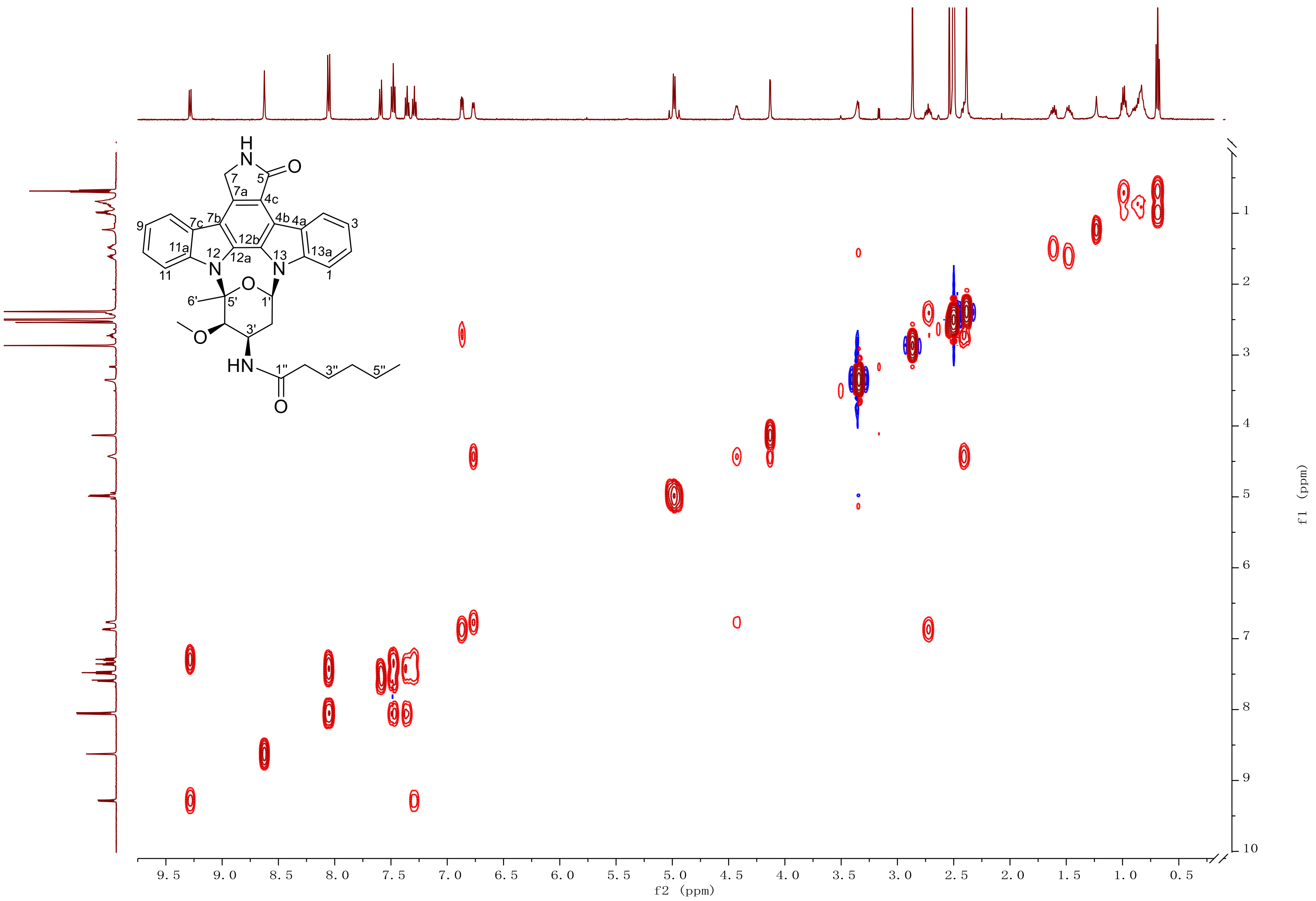

Figure S10. HMBC spectrum (500×125 MHz) of streptocarbazole F (**1**) in DMSO-d_6

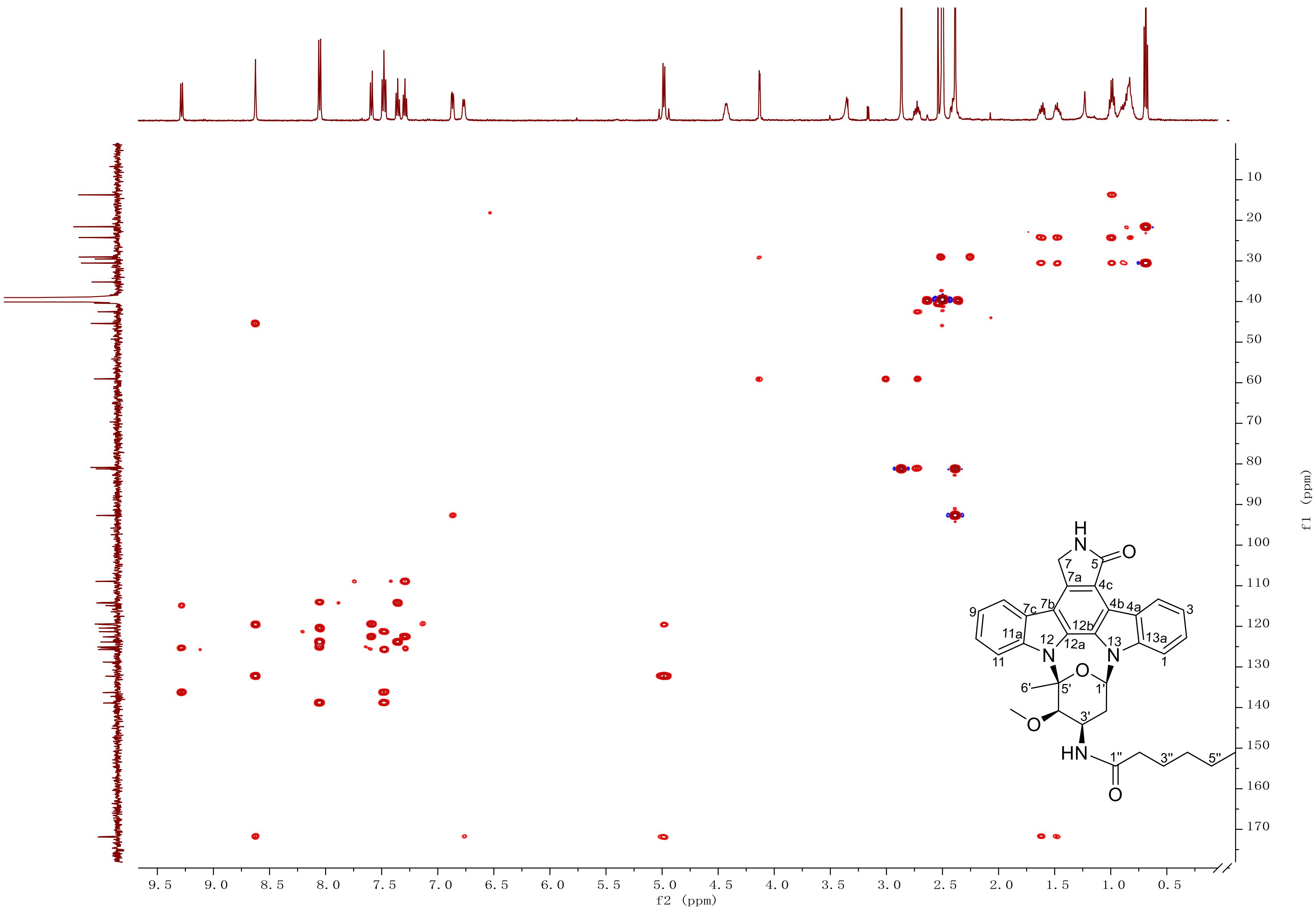

Figure S11. NOESY spectrum (500×500 MHz) of streptocarbazole F (**1**) in DMSO-d_6

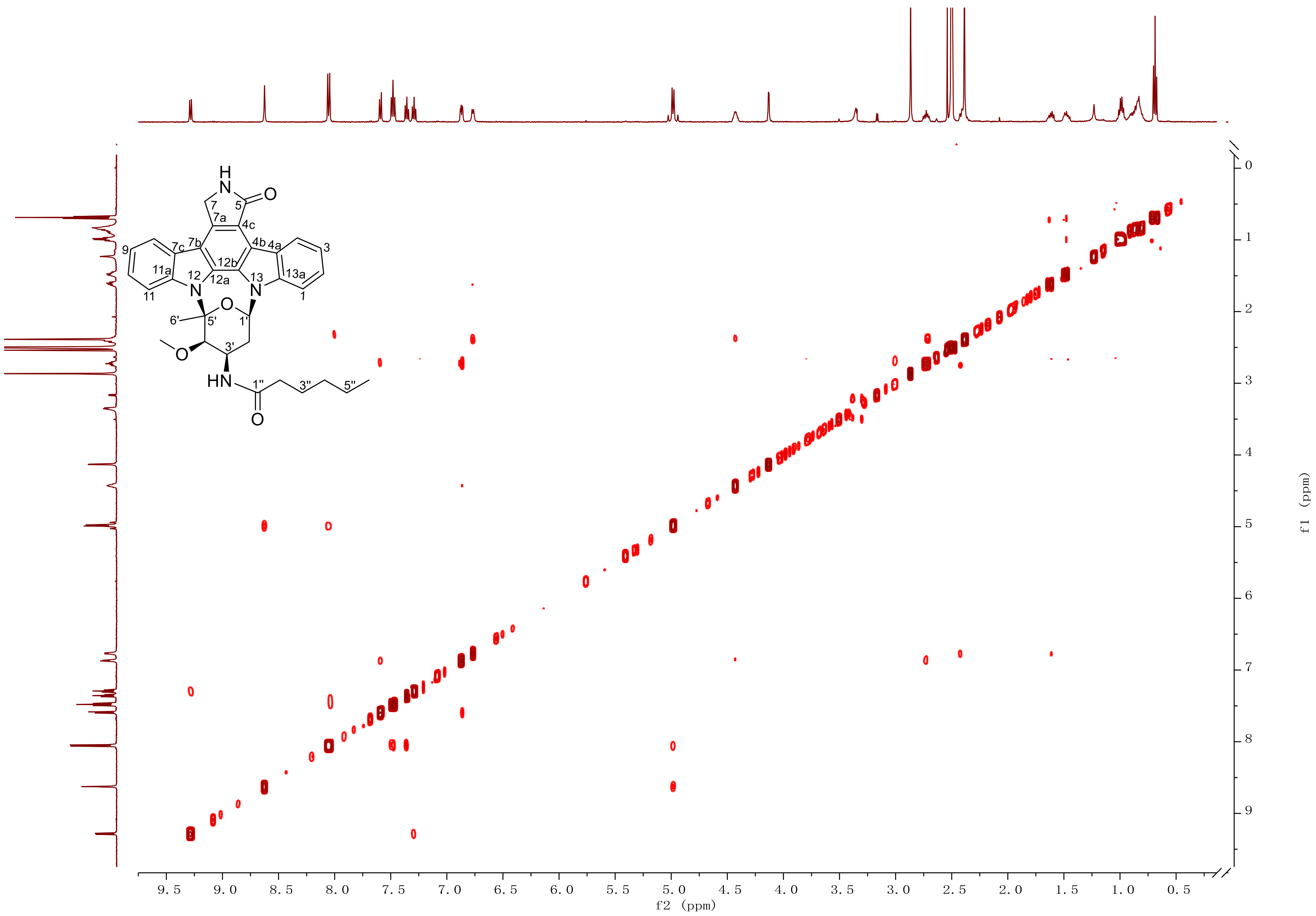

Figure S12. HRESIMS spectrum of streptocarbazole G (**2**)

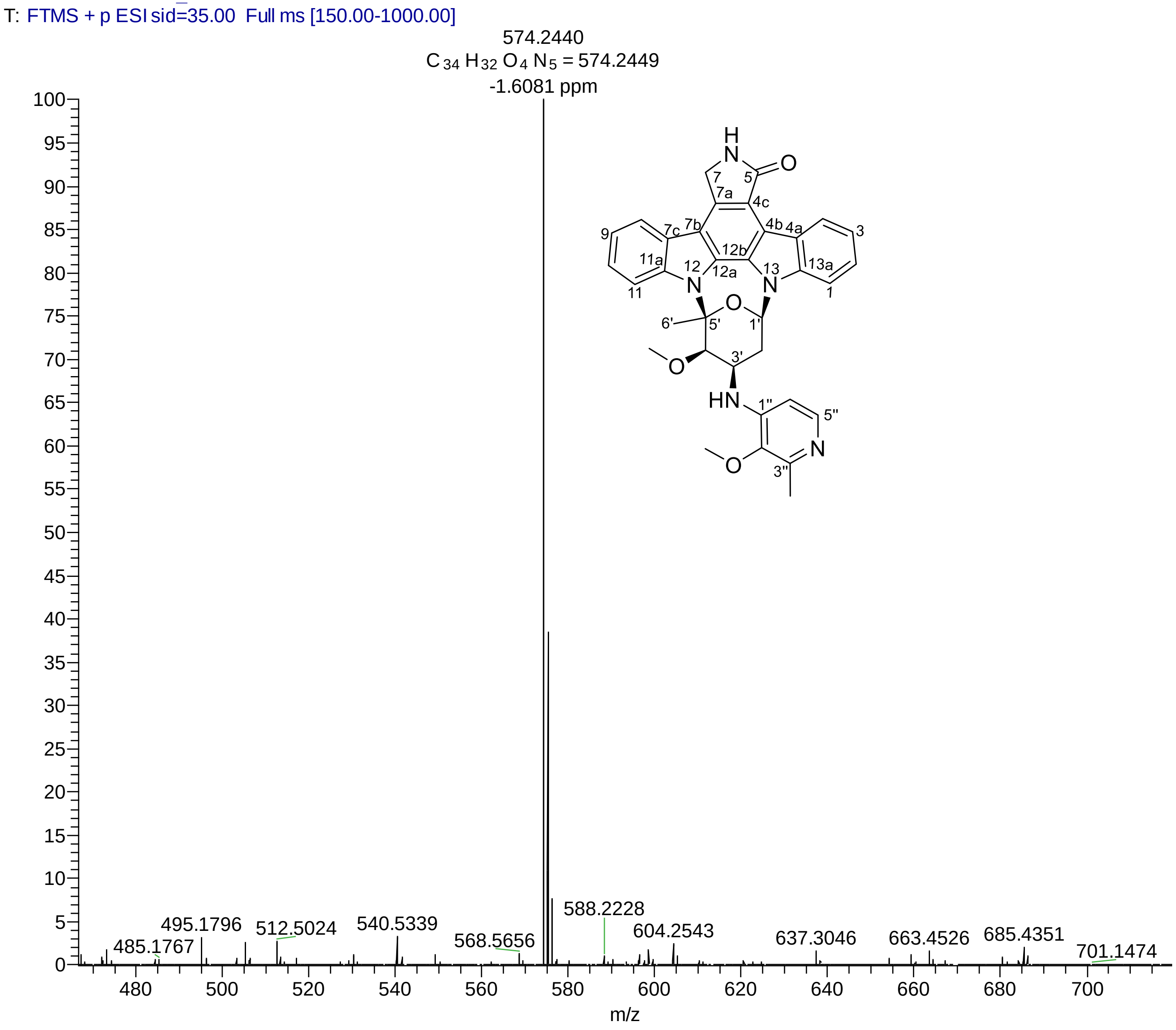

Figure S13. ^{1}H-NMR spectrum (500 MHz) of streptocarbazole G (**2**) in DMSO-d_6

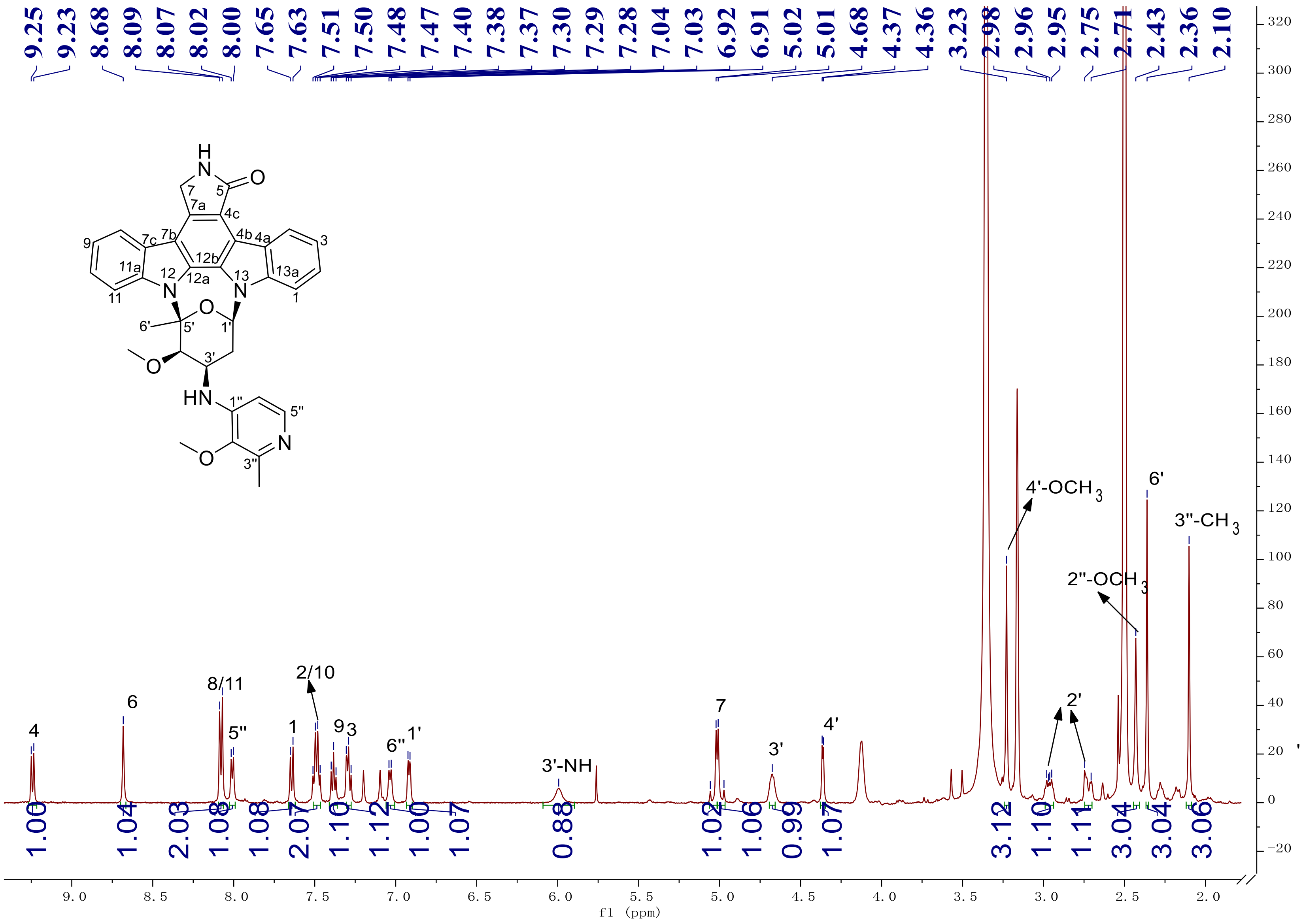

Figure S14. ^{13}C-NMR spectrum (125 MHz) of streptocarbazole G (**2**) in DMSO-d_6

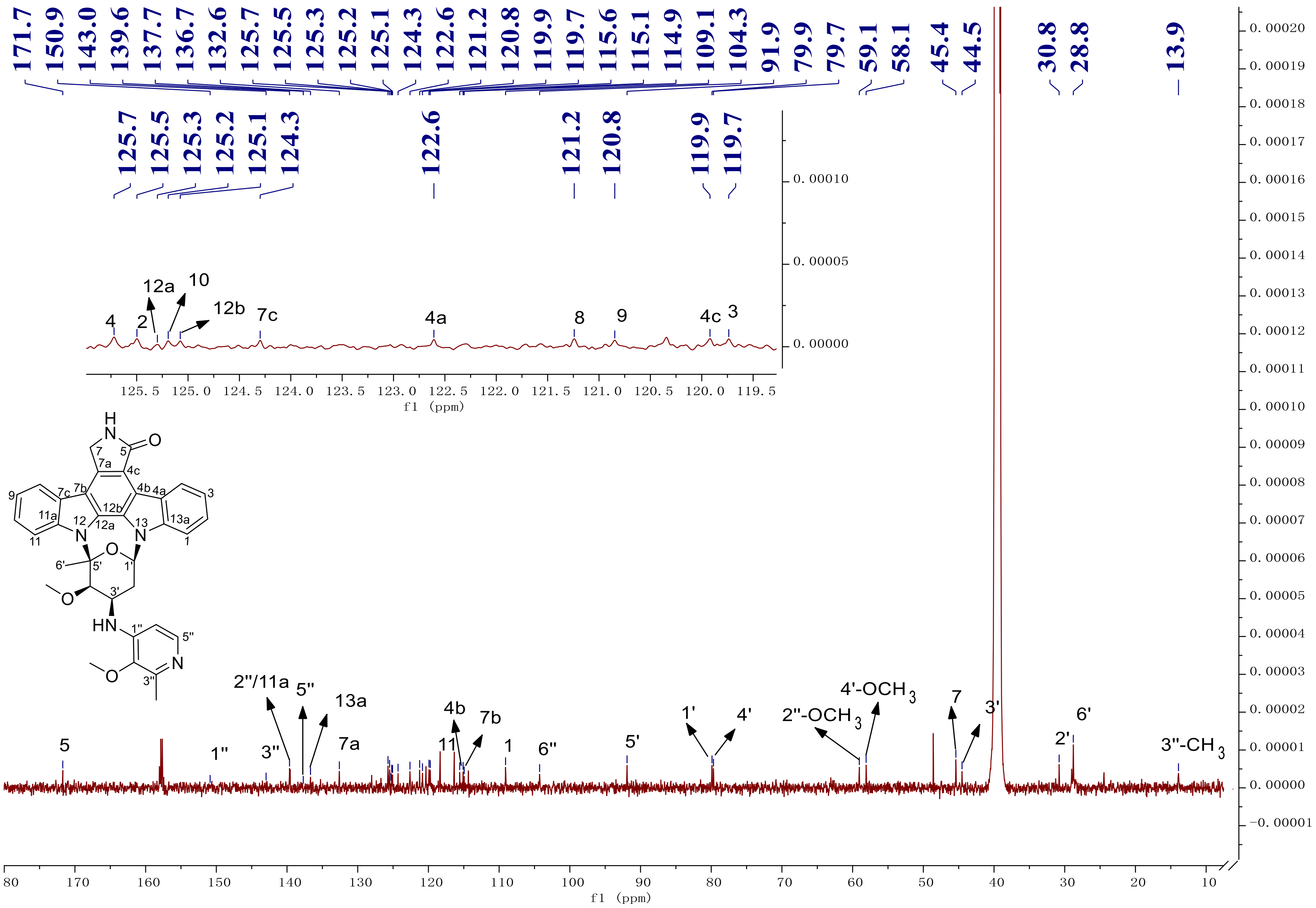

Figure S15. DEPT 135-NMR spectrum (125 MHz) of streptocarbazole G (**2**) in DMSO-d_6

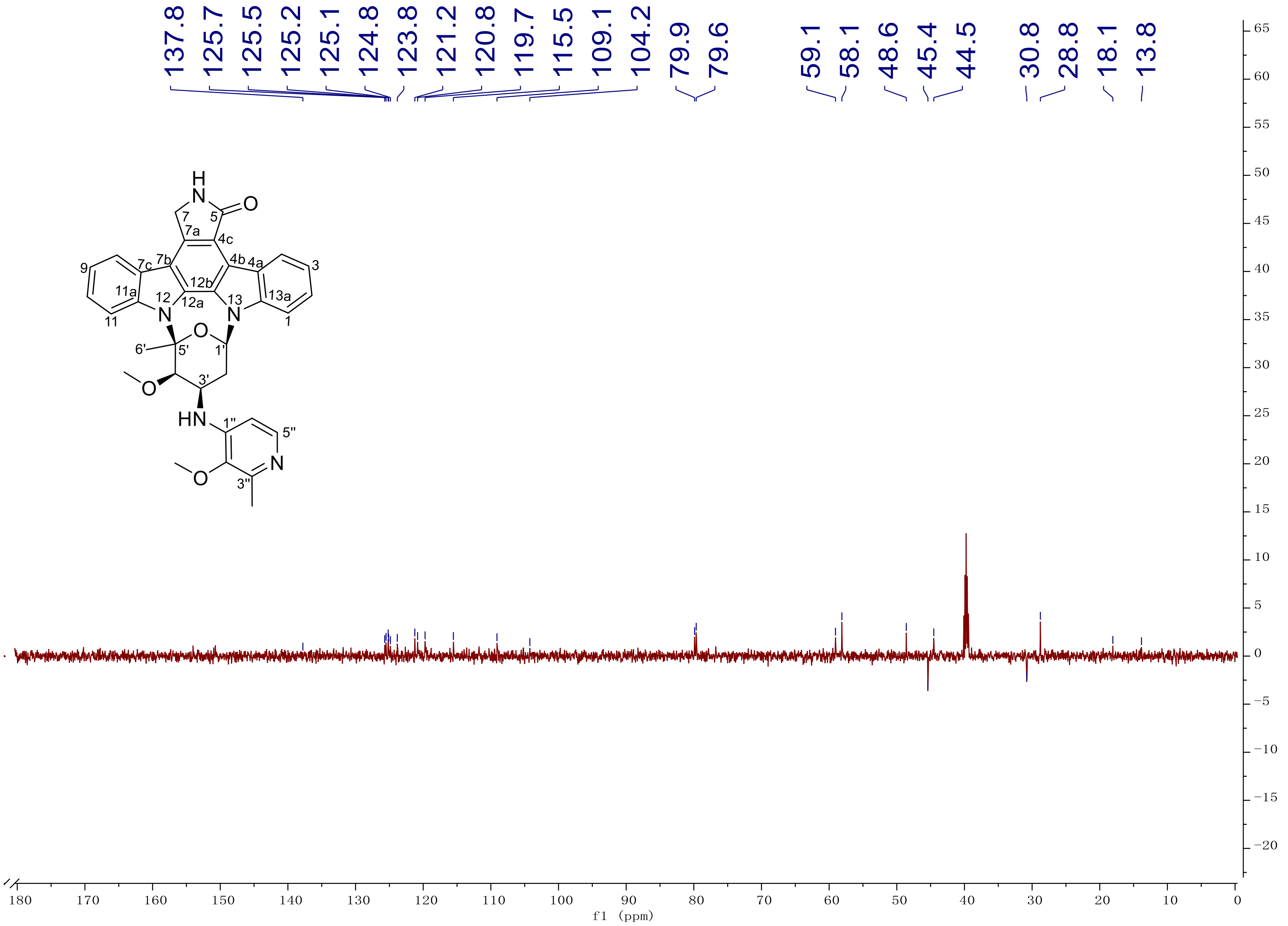

Figure S16. HSQC spectrum (500×125 MHz) of streptocarbazole G (**2**) in DMSO-d_6

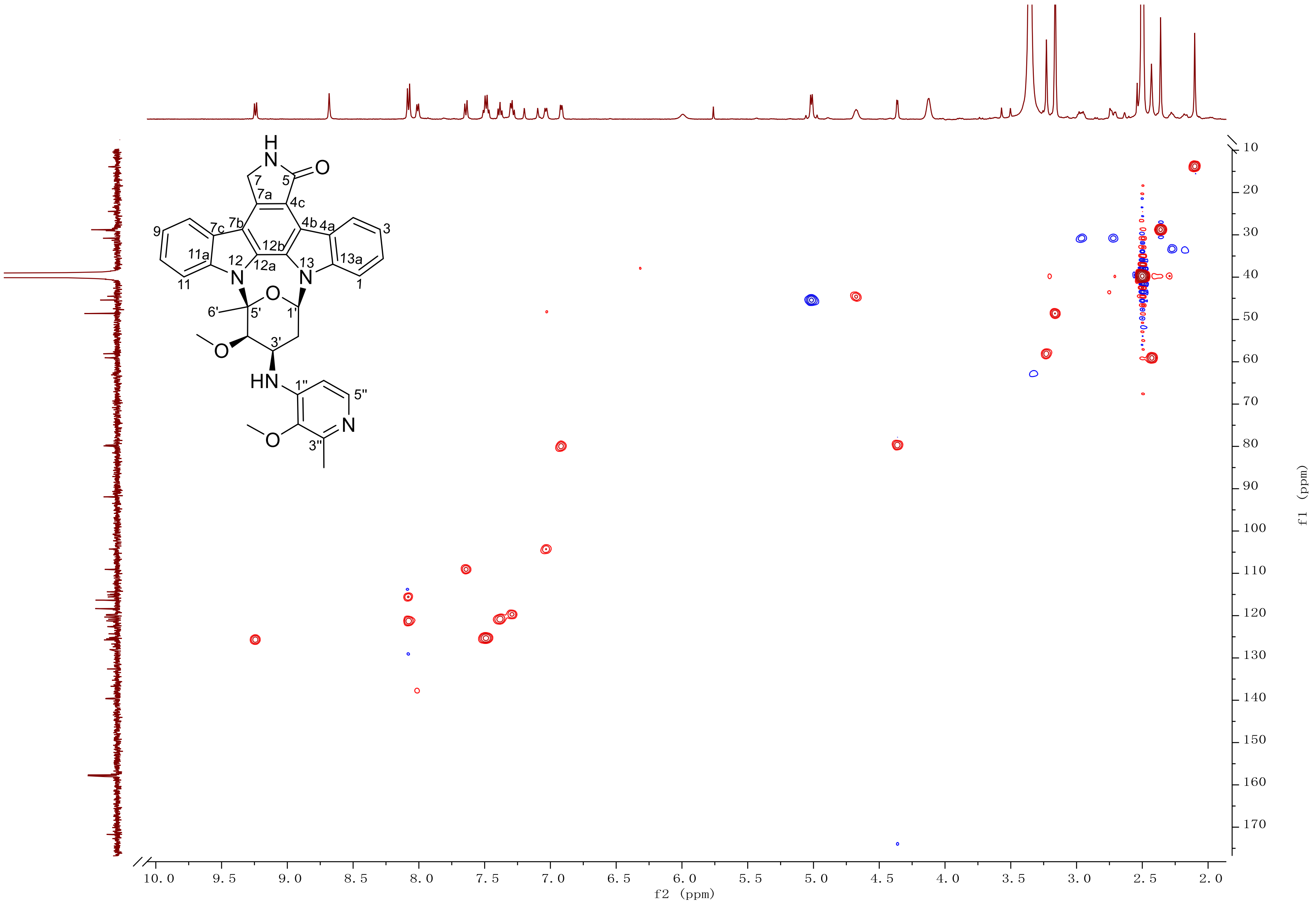

Figure S17. ^{1}H-^{1}H COSY spectrum (500×500 MHz) of streptocarbazole G (**2**) in DMSO-d_6

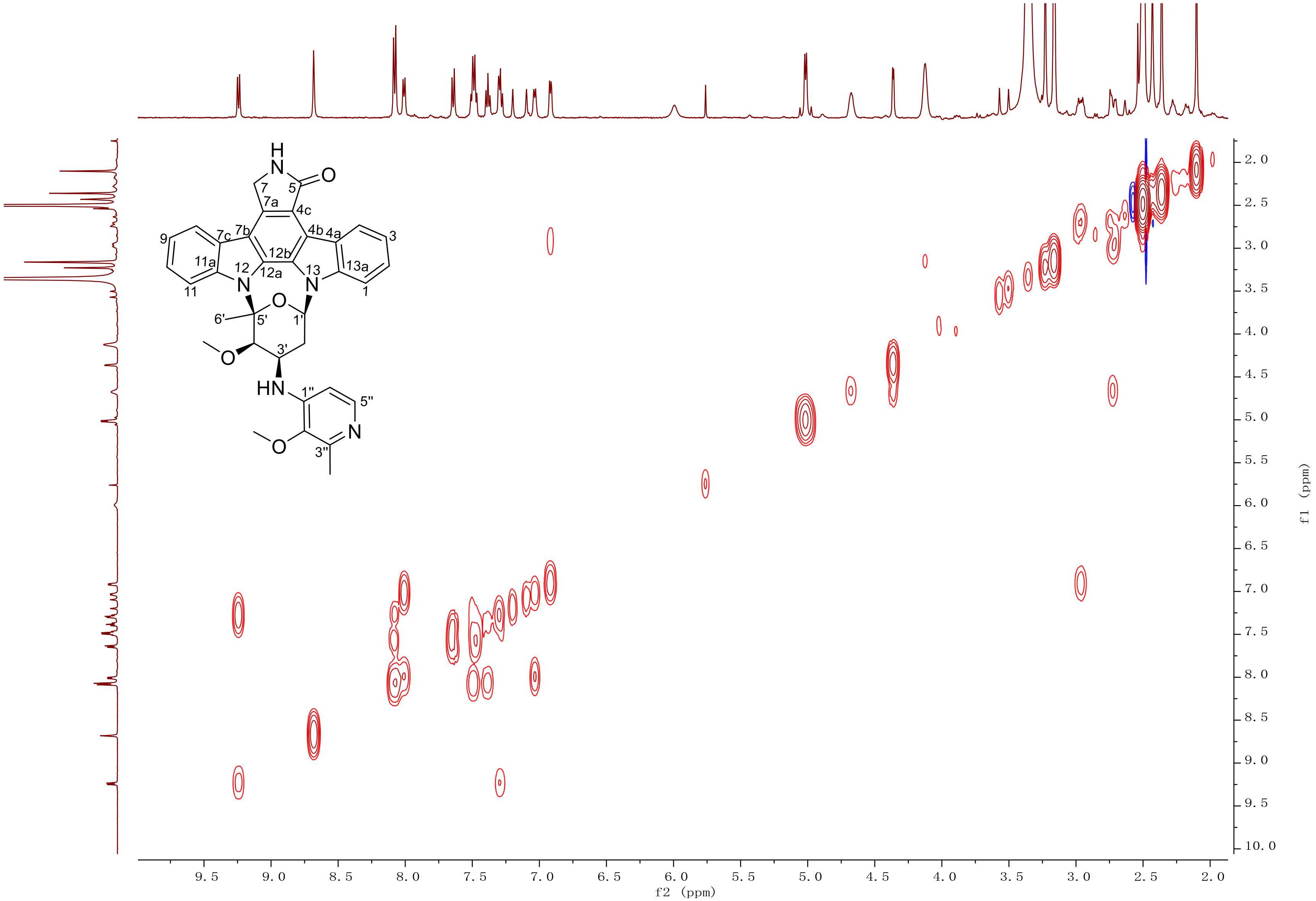

Figure S18. HMBC spectrum (500×125 MHz) of streptocarbazole G (**2**) in DMSO-d_6

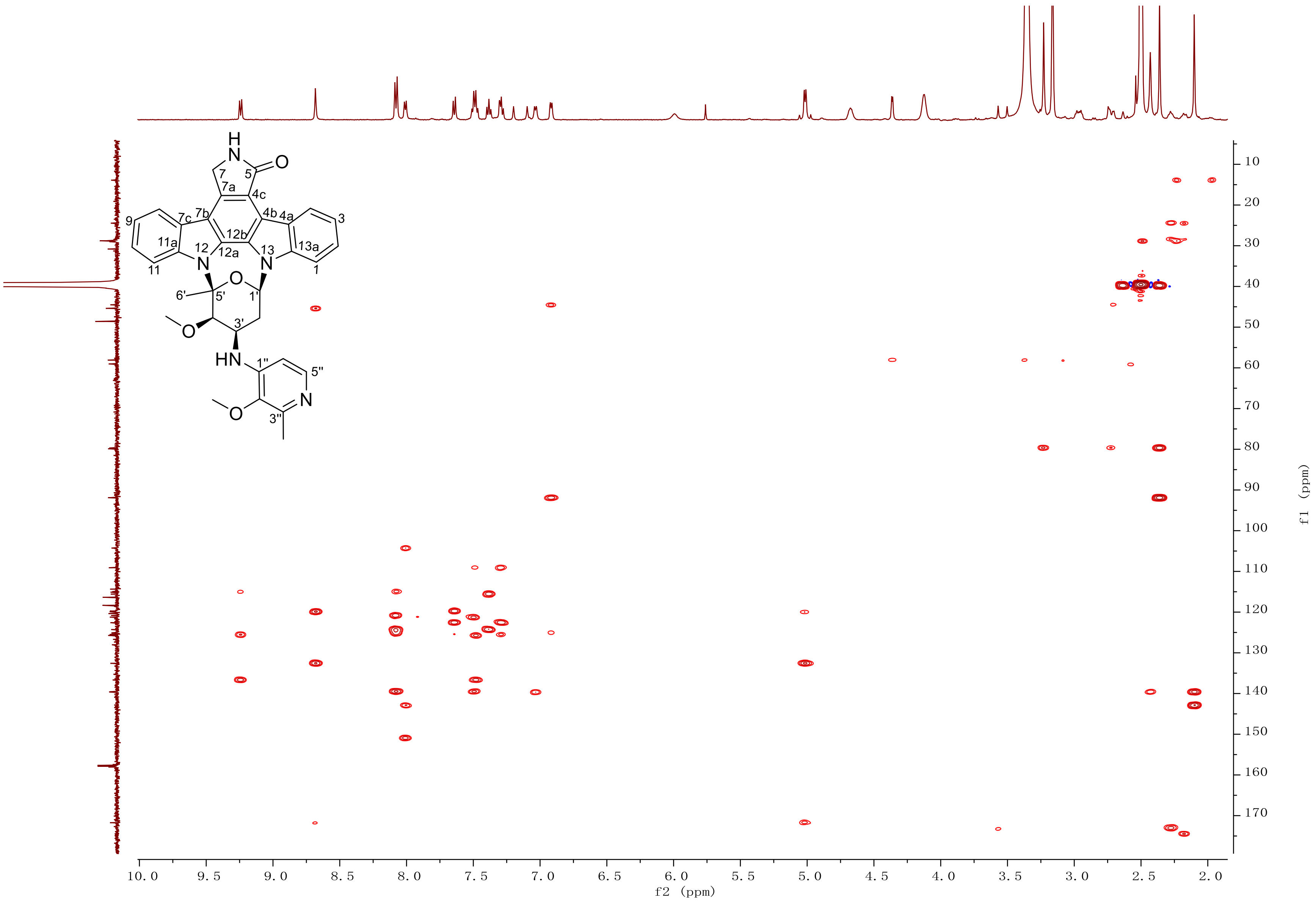

Figure S19. NOESY spectrum (500×500 MHz) of streptocarbazole G (**2**) in DMSO-d_6

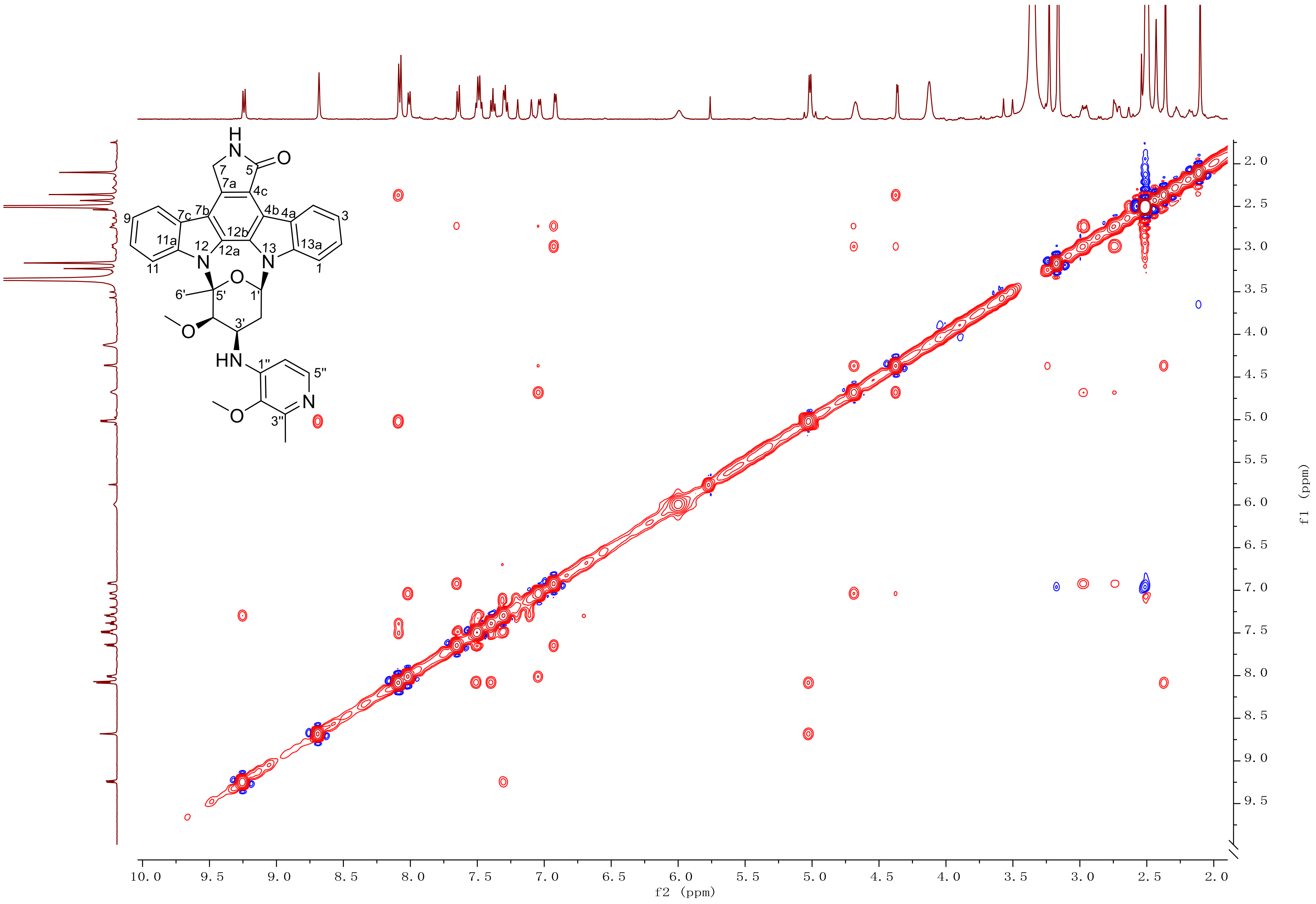

Figure S20. HRESIMS spectrum of streptocarbazole H (**3**)

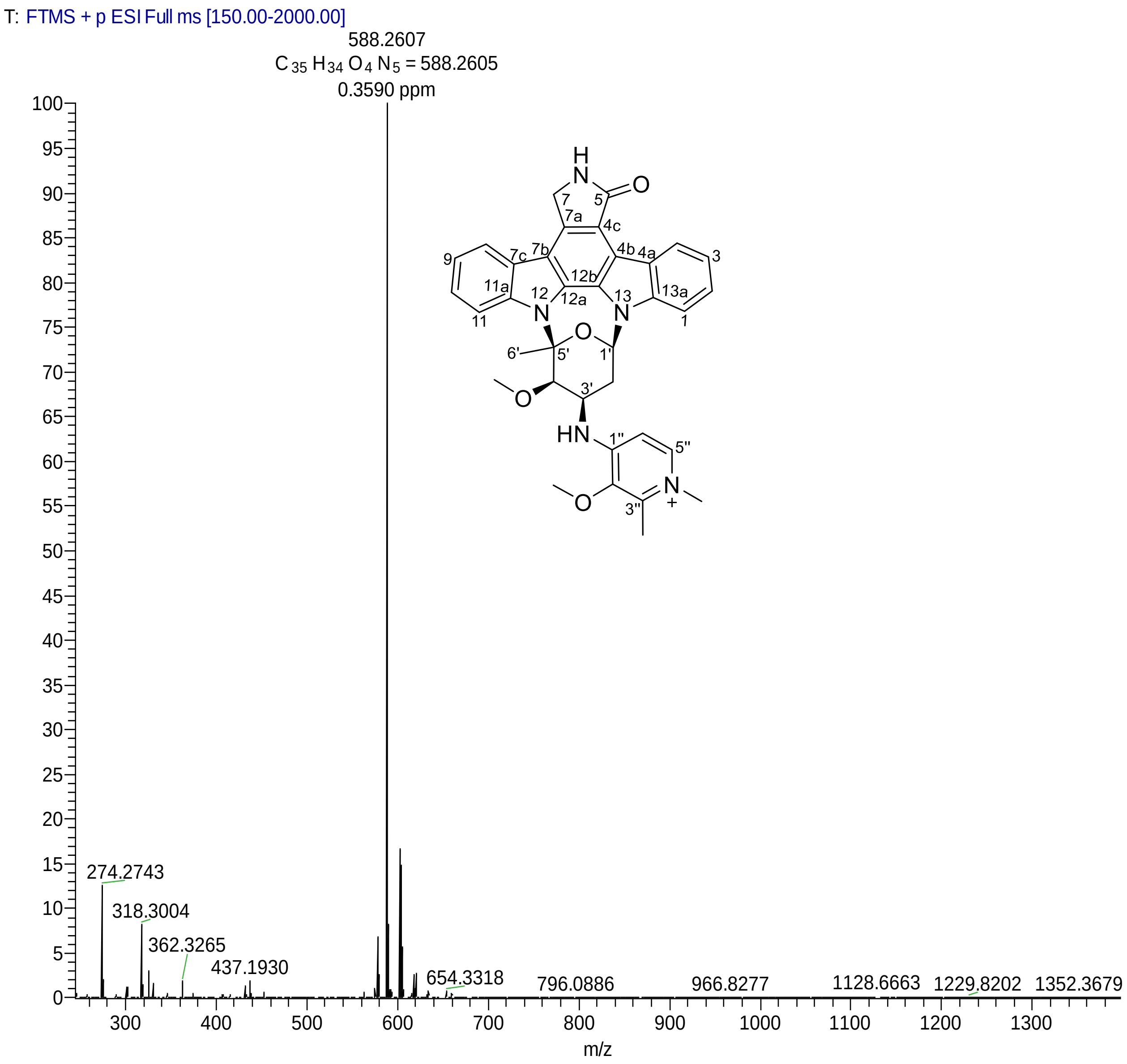

Figure S21. ^{1}H-NMR spectrum (600 MHz) of streptocarbazole H (**3**) in DMSO-d_6

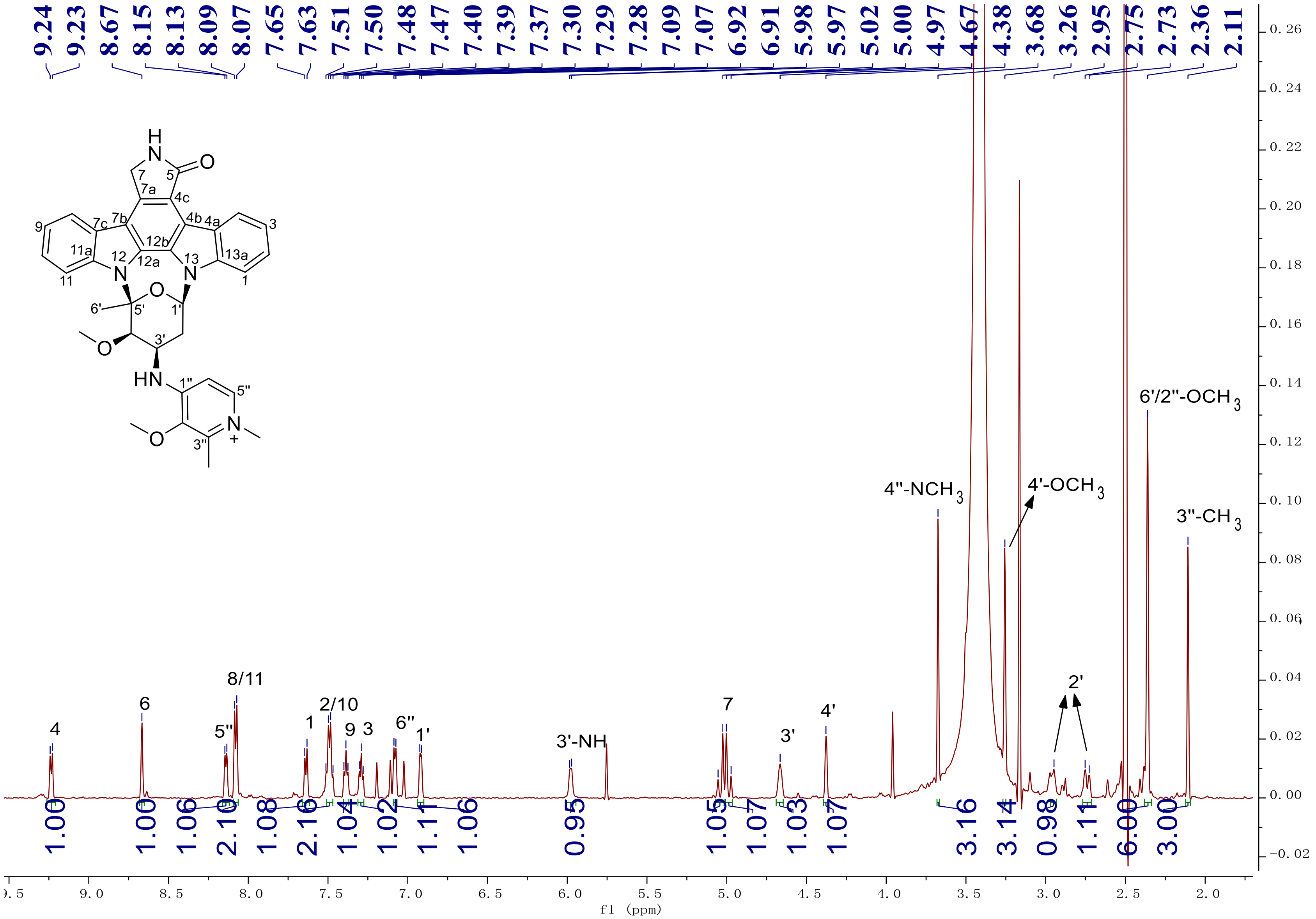

Figure S22. ^{13}C-NMR spectrum (150 MHz) of streptocarbazole H (**3**) in DMSO-d_6

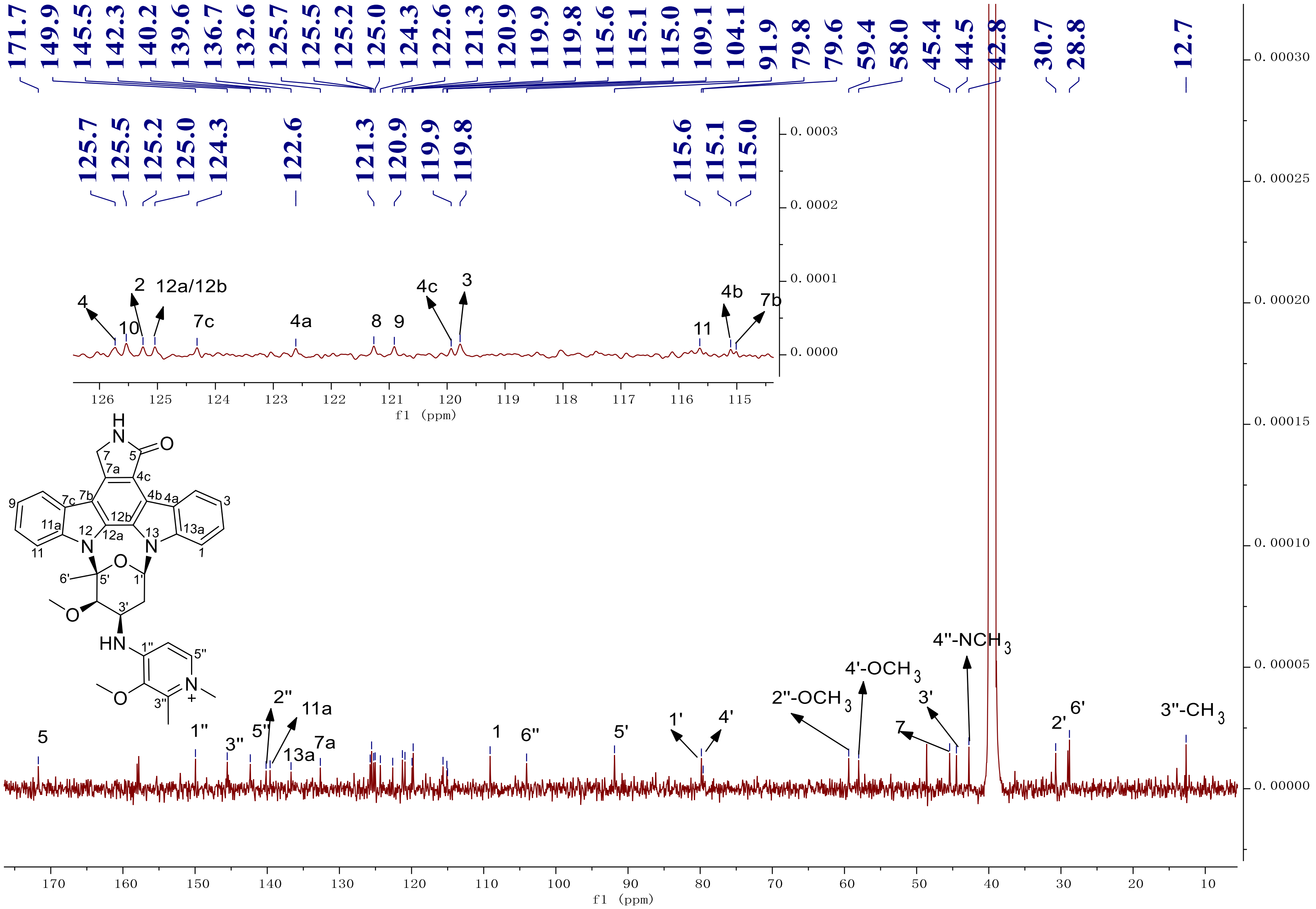

Figure S23. DEPT 135-NMR spectrum (150 MHz) of streptocarbazole H (**3**) in DMSO-d_6

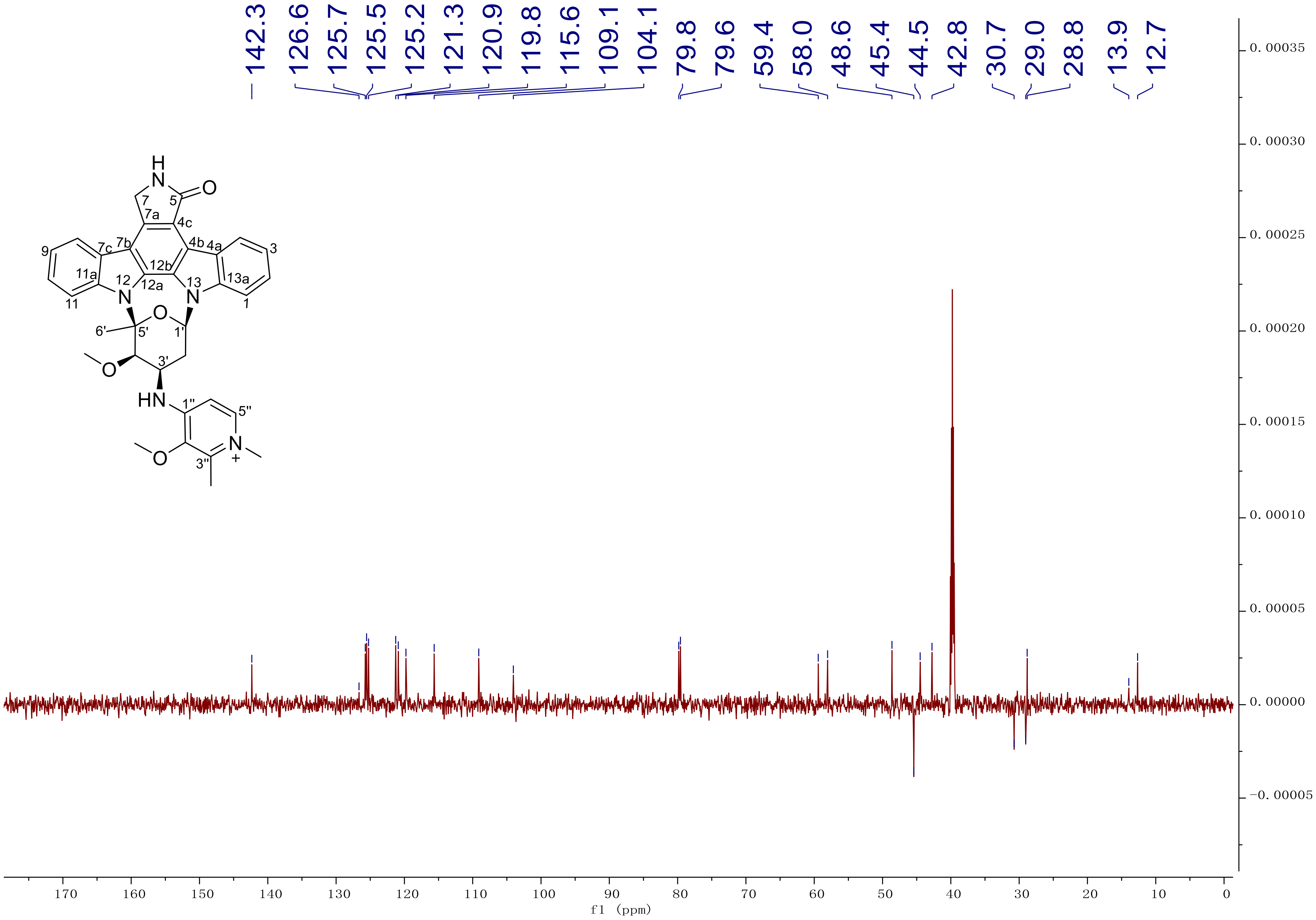

Figure S24. HSQC spectrum (600×150 MHz) of streptocarbazole H (**3**) in DMSO-d_6

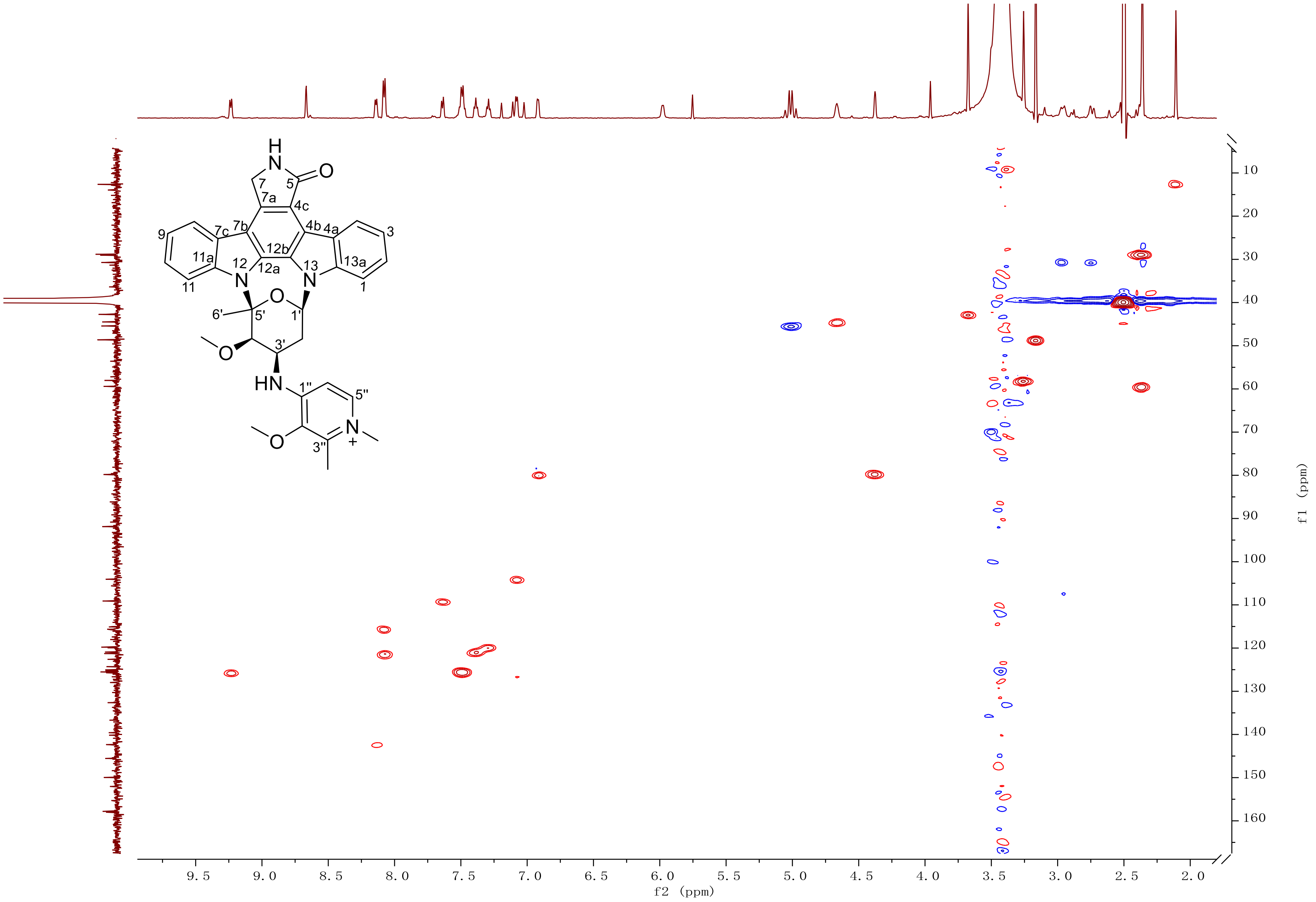

Figure S25. ^{1}H-^{1}H COSY spectrum (600×600 MHz) of streptocarbazole H (**3**) in DMSO-d_6

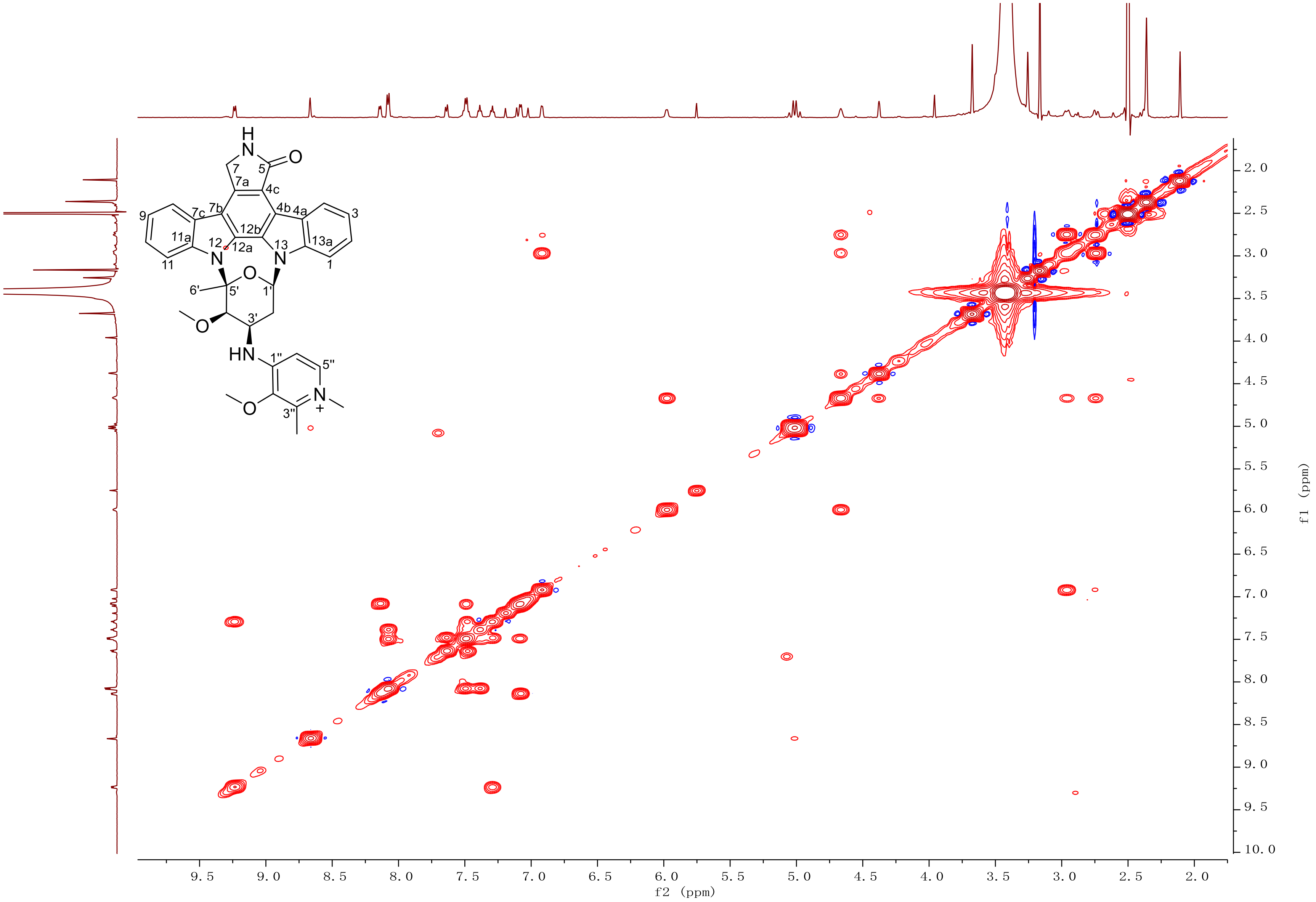

Figure S26. HMBC spectrum (600×150 MHz) of streptocarbazole H (**3**) in DMSO-d_6

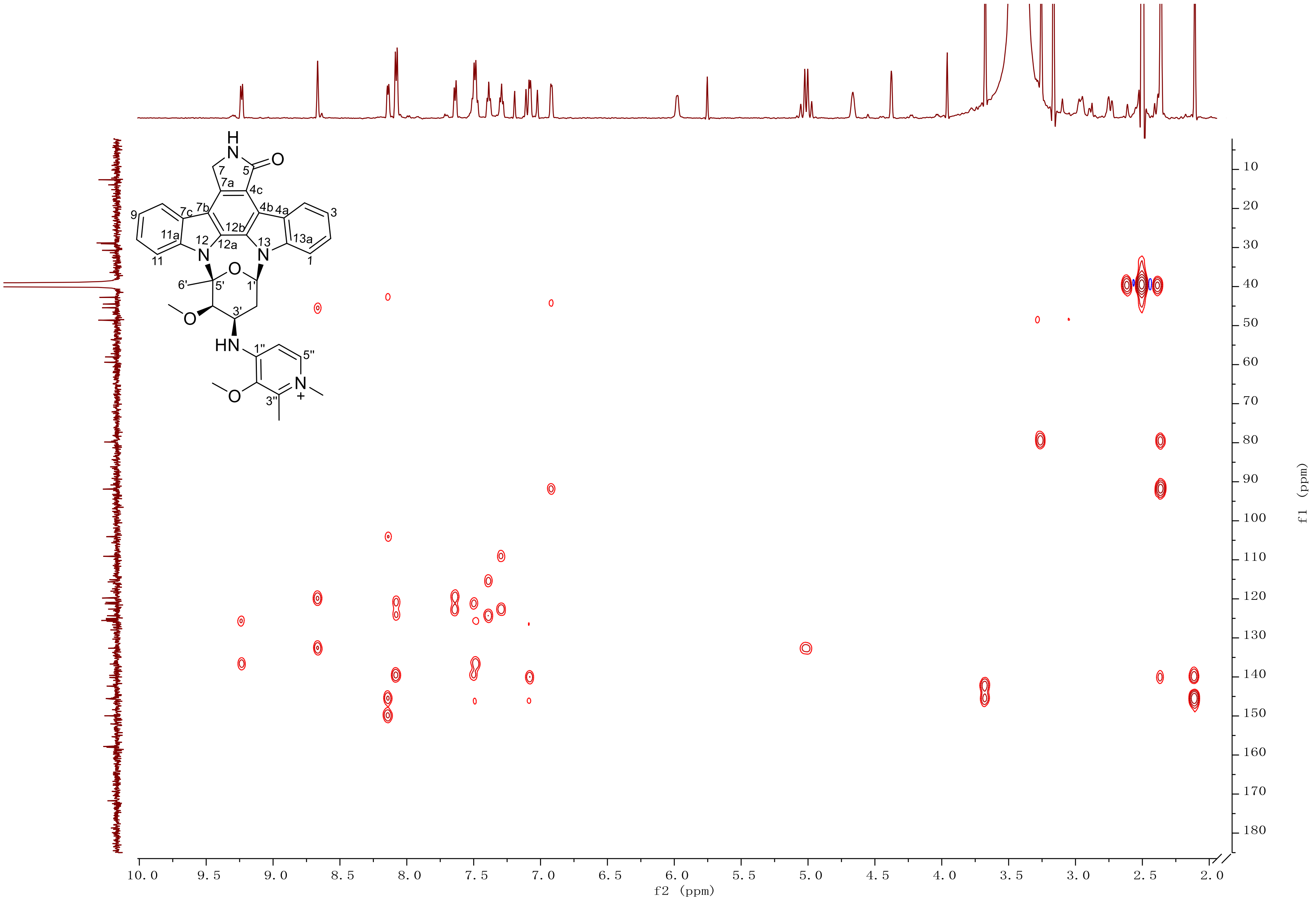

Figure S27. NOESY spectrum (600×600 MHz) of streptocarbazole H (**3**) in DMSO-d_6

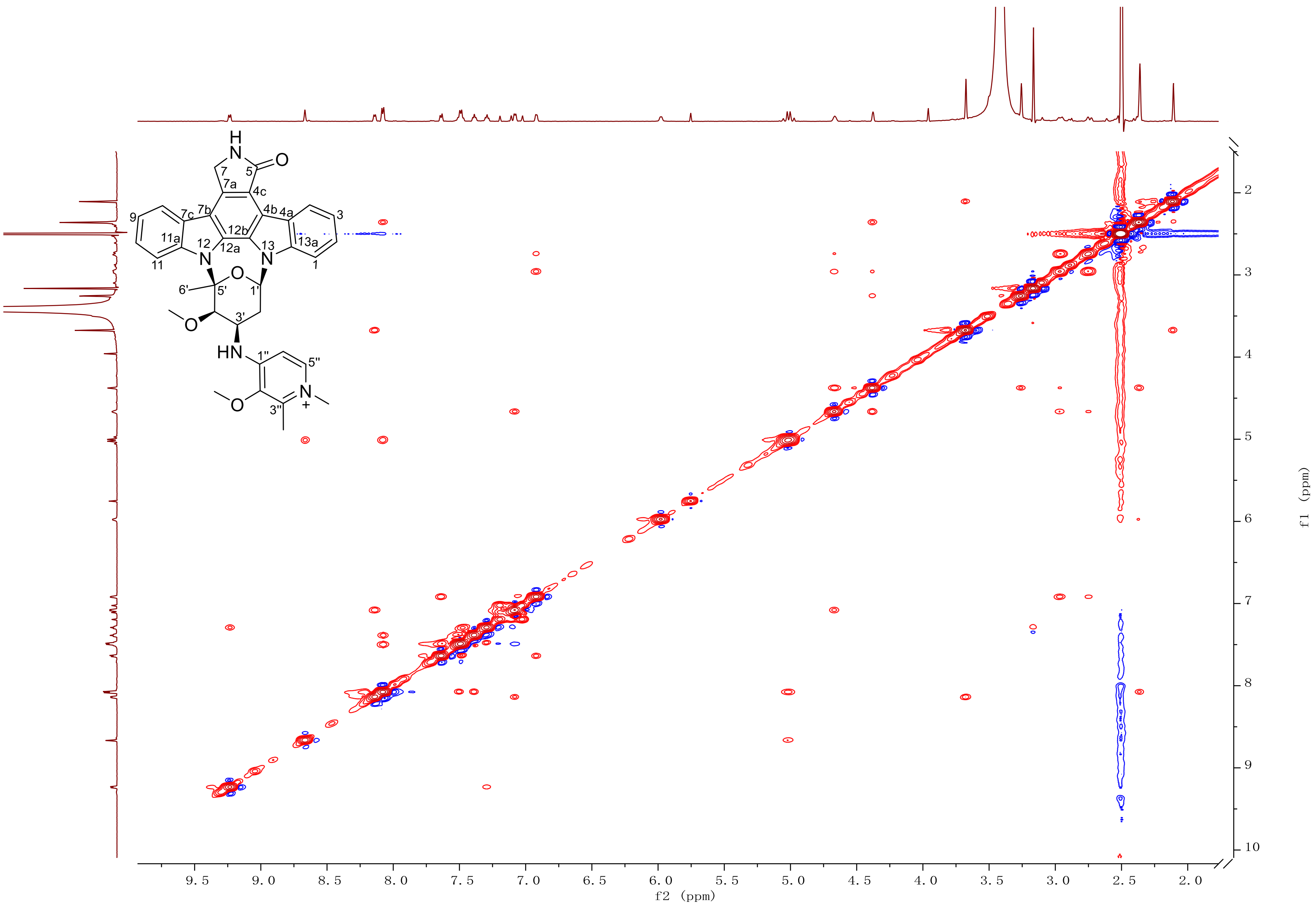

Figure S28. Experimental and calculated ECD curves for **1**

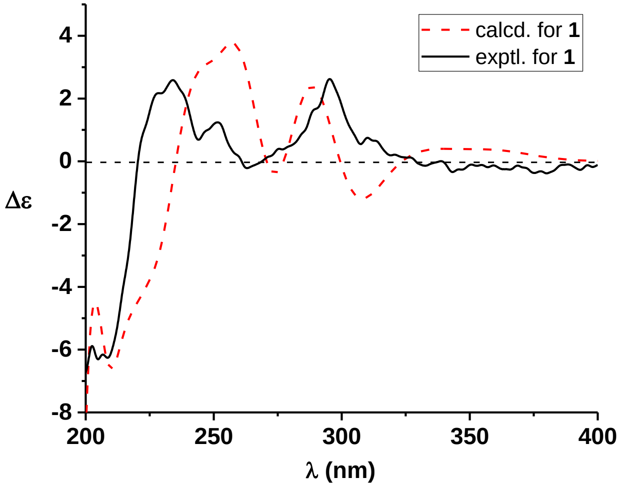

Theory and calculation details. The calculations were performed by using the density functional theory (DFT) as carried out in the Gaussian 09 (M. J. Frisch et al., 2010). Conformational searches were run by employing the "systematic" procedure implemented in Spartan'14 (Irvine CA., 2013) using MMFF. All MMFF minima were reoptimized with DFT calculations at the B3LYP/6-31G(d) level. Solvent effects of methanol solution were evaluated at the same DFT level by using the SCRF/PCM method (Miertus, S., 1982; Tomasi, J., 1994; Cammi, R., 1995). TDDFT (Casida, M. E., 1995; Gross, E. K. U., 1996; Gross, E. K. U., 1990; Runge, E., 1984) at B3LYP/6-31G(d) was employed to calculate the electronic excitation energies and rotational strengths in methanol. The overall calculated ECD curves were weighted by Boltzmann distribution (with a half-bandwidth of 0.20 eV) with a UV correction of 10 nm. The calculated ECD spectrum were produced by SpecDis 1.70.1 software (T. Bruhn, A., 2017).

References:

Gaussian 09, Revision B.01, M. J. Frisch, G. W. Trucks, H. B. Schlegel, G. E. Scuseria, M. A. Robb, J. R. Cheeseman, G. Scalmani, V. Barone, B. Mennucci, G. A. Petersson, H. Nakatsuji, M. Caricato, X. Li, H. P. Hratchian, A. F. Izmaylov, J. Bloino, G. Zheng, J. L. Sonnenberg, M. Hada, M. Ehara, K. Toyota, R. Fukuda, J. Hasegawa, M. Ishida, T. Nakajima, Y. Honda, O. Kitao, H. Nakai, T. Vreven, J. A. Montgomery, Jr., J. E. Peralta, F. Ogliaro, M. Bearpark, J. J. Heyd, E. Brothers, K. N. Kudin, V. N. Staroverov, T. Keith, R. Kobayashi, J. Normand, K. Raghavachari, A. Rendell, J. C. Burant, S. S. Iyengar, J. Tomasi, M. Cossi, N. Rega, J. M. Millam, M. Klene, J. E. Knox, J. B. Cross, V. Bakken, C. Adamo, J. Jaramillo, R. Gomperts, R. E. Stratmann, O. Yazyev, A. J. Austin, R. Cammi, C. Pomelli, J. W. Ochterski, R. L. Martin, K. Morokuma, V. G. Zakrzewski, G. A. Voth, P. Salvador, J. J. Dannenberg, S. Dapprich, A. D. Daniels, O. Farkas, J. B. Foresman, J. V. Ortiz, J. Cioslowski, and D. J. Fox, Gaussian, Inc., Wallingford CT, 2010.

Spartan'14, Wavefunction Inc., Irvine CA, 2013.

Miertus, S.; Tomasi, J. Chem. Phys. 1982, 65, 239–245.

Tomasi, J.; Persico, M. Chem. Rev. 1994, 94, 2027–2094.
Cammi, R.; Tomasi, J. J. Comp. Chem. 1995, 16, 1449–1458.
Casida, M. E. In Recent Advances in Density Functional Methods, part I; Chong, D. P., Eds.; World Scientific: Singapore, 1995; pp 155–192.
Gross, E. K. U.; Dobson, J. F.; Petersilka, M. Top. Curr. Chem. 1996, 181, 81–172.
Gross, E. K. U.; Kohn, W. Adv. Quantum Chem. 1990, 21, 255–291.
Runge, E.; Gross, E. K. U. Phys. Rev. Lett. 1984, 52, 997–1000.
T. Bruhn, A. Schaumlöffel, Y. Hemberger. G. Pescitelli, SpecDis, Version 1.70.1, Berlin, Germany, 2017, https://specdis-software.jimdo.com.

Table S2. DFT-optimized structures and thermodynamic parameters for low-energy conformers of **1**

Conformers	DFT-optimized structures	Population (%)	Total energy (a.u.)	Sum of electronic and zero-point energies (a.u.)	Sum of electronic and thermal energies (a.u.)	Sum of electronic and thermal enthalpies (a.u.)	Sum of electronic and thermal free energies (a.u.)
Conf. A	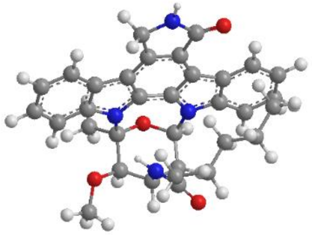	41.0	-1797.96496134	-1797.341140	-1797.306002	-1797.305057	-1797.406955
Conf. B	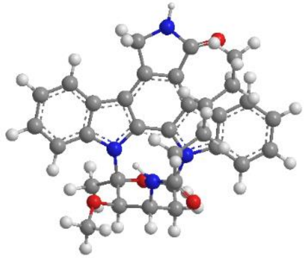	26.6	-1797.96455399	-1797.341214	-1797.305765	-1797.304821	-1797.408279

Conf. C	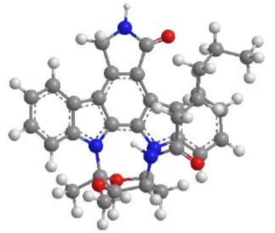	7.8	-1797.96339916	-1797.339721	-1797.304385	-1797.303440	-1797.406881
Conf. D	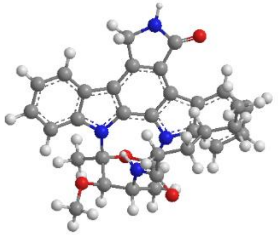	7.1	-1797.96331257	-1797.339792	-1797.304429	-1797.303484	-1797.407566
Conf. E	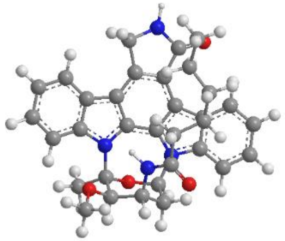	6.7	-1797.96325685	-1797.340041	-1797.304487	-1797.303543	-1797.408791
Conf. F	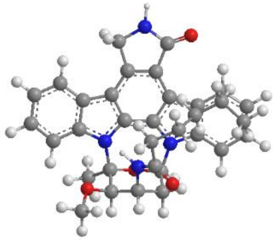	6.4	-1797.96320969	-1797.339842	-1797.304438	-1797.303494	-1797.408499
Conf. G	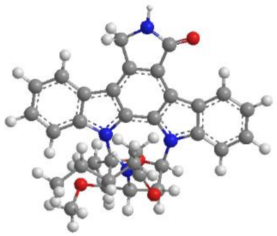	4.3	-1797.96282745	-1797.339574	-1797.304117	-1797.303173	-1797.408387

Table S3. Optimized Z-matrixes of **1** in the gas phase (Å) at B3LYP/6-31G(d) level

Conf. A				Conf. B				Conf. C				Conf. D			
C	-4.10096	3.681915	0.7942	C	-3.48659	4.252388	0.15465	C	-3.96091	3.866205	0.547392	C	-4.29956	3.381383	1.261987
C	-4.87958	2.531382	0.594915	C	-4.43593	3.224608	0.265365	C	-4.78946	2.733296	0.543469	C	-5.0068	2.195319	1.011281
C	-4.32887	1.352198	0.099124	C	-4.10743	1.894853	0.0141	C	-4.31351	1.480117	0.166319	C	-4.40327	1.099507	0.399251
C	-2.96516	1.33309	-0.22328	C	-2.79682	1.590911	-0.37762	C	-2.9763	1.364311	-0.23615	C	-3.06129	1.204598	0.011002
C	-2.16477	2.484836	0.006216	C	-1.82278	2.622421	-0.45911	C	-2.12359	2.50058	-0.20165	C	-2.33051	2.390507	0.290878
C	-2.74095	3.658527	0.512025	C	-2.17612	3.95337	-0.19577	C	-2.62423	3.750879	0.187567	C	-2.95806	3.478149	0.914173
N	-2.14312	0.299831	-0.72819	N	-2.18037	0.357057	-0.68508	N	-2.22551	0.244479	-0.65876	N	-2.19934	0.27304	-0.61184
C	-0.83299	0.798542	-0.79375	C	-0.82413	0.613901	-0.9345	C	-0.90607	0.673549	-0.86331	C	-0.9314	0.868371	-0.7006
C	-0.80891	2.140645	-0.34148	C	-0.5672	2.000001	-0.79566	C	-0.80561	2.058242	-0.58186	C	-0.97454	2.171186	-0.14677
C	0.373151	0.174846	-1.14963	C	0.242909	-0.25802	-1.20348	C	0.252028	-0.04203	-1.20847	C	0.293401	0.357676	-1.15796
C	1.609035	0.869451	-1.07386	C	1.571273	0.227848	-1.33052	C	1.518091	0.598546	-1.26651	C	1.483144	1.128425	-1.07951
C	1.604251	2.20554	-0.6212	C	1.799121	1.613037	-1.18713	C	1.589749	1.978064	-0.97843	C	1.409498	2.429633	-0.53907
C	0.413697	2.827359	-0.26628	C	0.744763	2.480653	-0.93227	C	0.444773	2.693057	-0.65131	C	0.199783	2.937997	-0.08284
N	0.604589	-1.11929	-1.57971	N	0.245918	-1.63389	-1.3484	N	0.408717	-1.38625	-1.49676	N	0.58839	-0.88564	-1.69035
C	1.965881	-1.27952	-1.81119	C	1.548069	-2.0574	-1.58884	C	1.750482	-1.63555	-1.76136	C	1.947926	-0.93701	-1.97911
C	2.628944	-0.06039	-1.5063	C	2.407546	-0.92577	-1.58161	C	2.478122	-0.42256	-1.62501	C	2.54363	0.298525	-1.6074
C	2.660036	-2.40387	-2.26997	C	2.030995	-3.35045	-1.81436	C	2.375694	-2.83629	-2.11304	C	2.695492	-1.97824	-2.53893
C	4.038255	-2.28627	-2.43217	C	3.397273	-3.49558	-2.04167	C	3.7499	-2.804	-2.33647	C	4.05872	-1.76229	-2.72675
C	4.709937	-1.08599	-2.1387	C	4.262052	-2.38661	-2.03659	C	4.485118	-1.61227	-2.20578	C	4.664627	-0.54716	-2.36055
C	4.019001	0.029327	-1.67497	C	3.7815	-1.10157	-1.80692	C	3.863304	-0.41981	-1.85	C	3.920492	0.486942	-1.80074
C	2.729912	3.152249	-0.39248	C	3.067249	2.391132	-1.21185	C	2.763791	2.889061	-0.90363	C	2.47202	3.452281	-0.3312
N	2.135776	4.319722	0.040236	N	2.686959	3.706806	-1.04274	N	2.238967	4.131606	-0.61264	N	1.82721	4.527049	0.245636
C	0.694493	4.239094	0.194343	C	1.266034	3.893591	-0.8092	C	0.80552	4.135207	-0.37975	C	0.403945	4.335906	0.4537
O	3.936609	2.984776	-0.53031	O	4.223287	2.002051	-1.33604	O	3.956789	2.643307	-1.04423	O	3.667895	3.40605	-0.593
C	-2.58901	-0.9726	-1.37482	C	-2.85709	-0.93841	-0.99699	C	-2.75088	-1.08319	-1.09761	C	-2.59594	-0.95649	-1.36472
C	-2.88037	-2.09375	-0.33257	C	-3.19259	-1.75289	0.289671	C	-2.98322	-2.04994	0.103644	C	-2.76989	-2.18929	-0.42788
C	-1.617	-2.67649	0.322536	C	-1.96876	-2.40198	0.956302	C	-1.68937	-2.62196	0.706522	C	-1.44503	-2.76633	0.095925
C	-0.66386	-3.13775	-0.79222	C	-1.23109	-3.2362	-0.10321	C	-0.87184	-3.26158	-0.42742	C	-0.53483	-3.05756	-1.10828
C	-0.4247	-2.09478	-1.8898	C	-0.93818	-2.47526	-1.39997	C	-0.6728	-2.34649	-1.6395	C	-0.39554	-1.88246	-2.08185
O	-1.60308	-1.41373	-2.29911	O	-2.03655	-1.70621	-1.86906	O	-1.85368	-1.66408	-2.03589	O	-1.62725	-1.23448	-2.36864
C	-3.80489	-0.73086	-2.27906	C	-4.11701	-0.70926	-1.84276	C	-4.03365	-0.92088	-1.9244	C	-3.8662	-0.70923	-2.18892
O	-3.80287	-1.5691	0.60094	O	-3.91076	-0.89204	1.149552	O	-3.78218	-1.359	1.042349	O	-3.66458	-1.81273	0.598171
N	-1.03698	-1.72844	1.262749	N	-1.13639	-1.40405	1.611192	N	-0.96791	-1.60655	1.458523	N	-0.84515	-1.90029	1.09955
C	-4.41972	-2.53203	1.443525	C	-4.56429	-1.53203	2.236204	C	-4.35373	-2.16864	2.06008	C	-4.17967	-2.88542	1.373188
C	-0.14178	-2.13268	2.216766	C	-0.15592	-1.76741	2.495778	C	0.059809	-1.94168	2.299411	C	0.105689	-2.37975	1.958681

C	0.199611	-1.08657	3.275494	C	0.638434	-0.62288	3.109888	C	0.717509	-0.78286	3.035712	C	0.5632	-1.41205	3.046884
C	1.673654	-1.11798	3.702838	C	2.087525	-0.61431	2.585278	C	2.168297	-0.57512	2.558704	C	2.068907	-1.52098	3.330544
C	2.64288	-0.62111	2.622727	C	2.939285	0.509585	3.186835	C	2.875703	0.568362	3.297892	C	2.941506	-1.02375	2.169848
C	4.095018	-0.53667	3.110803	C	4.357445	0.5683	2.602476	C	4.272017	0.908246	2.748627	C	4.454277	-1.14836	2.413823
C	5.067322	-0.04414	2.033768	C	5.218401	1.671362	3.226421	C	5.301309	-0.21864	2.89526	C	4.995218	-0.24794	3.531238
O	0.323698	-3.26897	2.224611	O	0.09192	-2.94691	2.733655	O	0.448063	-3.10192	2.411272	O	0.542989	-3.5235	1.857692
H	-4.56011	4.585732	1.184291	H	-3.77415	5.279636	0.359414	H	-4.36217	4.829671	0.848327	H	-4.79818	4.217735	1.743434
H	-5.93711	2.549913	0.844277	H	-5.45192	3.463722	0.56782	H	-5.82647	2.826432	0.854594	H	-6.04813	2.118242	1.312226
H	-4.94003	0.465525	0.016995	H	-4.84358	1.120572	0.171091	H	-4.95856	0.616818	0.236547	H	-4.95357	0.1781	0.277463
H	-2.12945	4.539568	0.684012	H	-1.43206	4.741927	-0.26187	H	-1.97282	4.619712	0.210017	H	-2.40029	4.385777	1.125916
H	2.15515	-3.34052	-2.48724	H	1.376127	-4.21669	-1.80593	H	1.822043	-3.76627	-2.20239	H	2.242547	-2.92707	-2.81111
H	4.603419	-3.14352	-2.78814	H	3.800581	-4.48887	-2.21972	H	4.262054	-3.72237	-2.61085	H	4.664235	-2.55416	-3.1595
H	5.786388	-1.03079	-2.27414	H	5.323484	-2.53801	-2.21148	H	5.55695	-1.62538	-2.38223	H	5.731533	-0.4153	-2.51703
H	4.523358	0.959941	-1.4396	H	4.43675	-0.23755	-1.79588	H	4.41757	0.505939	-1.74125	H	4.372282	1.430863	-1.51558
H	2.695653	5.106698	0.331366	H	3.379763	4.431336	-0.92927	H	2.847205	4.909995	-0.40758	H	2.330418	5.369391	0.479015
H	0.177063	4.988214	-0.42296	H	0.817249	4.568638	-1.55257	H	0.285596	4.828311	-1.05721	H	-0.19233	5.08099	-0.09393
H	0.385866	4.402725	1.237947	H	1.068515	4.321035	0.185564	H	0.560775	4.435901	0.650279	H	0.132816	4.415224	1.517256
H	-3.34804	-2.91526	-0.89993	H	-3.84235	-2.57996	-0.04131	H	-3.54243	-2.90747	-0.30564	H	-3.22522	-2.97582	-1.05257
H	-1.89231	-3.57771	0.885182	H	-2.3121	-3.10653	1.724111	H	-1.94823	-3.4319	1.399903	H	-1.64159	-3.73259	0.575224
H	-1.10992	-4.02188	-1.26615	H	-1.8642	-4.09648	-0.35699	H	-1.40952	-4.15549	-0.76927	H	-0.96804	-3.89944	-1.66385
H	0.287523	-3.44732	-0.35331	H	-0.30402	-3.62692	0.32284	H	0.095139	-3.58874	-0.03807	H	0.449372	-3.37249	-0.75307
H	-0.09482	-2.61396	-2.796	H	-0.76686	-3.20281	-2.20075	H	-0.4242	-2.966	-2.50802	H	-0.07192	-2.27089	-3.05372
H	-3.58326	0.084827	-2.9716	H	-3.87006	-0.08035	-2.70158	H	-3.86447	-0.19119	-2.71999	H	-3.73188	0.184786	-2.80274
H	-3.98076	-1.64129	-2.85789	H	-4.4566	-1.68135	-2.20946	H	-4.26205	-1.88718	-2.38145	H	-4.01128	-1.56734	-2.85037
H	-4.70614	-0.49159	-1.71949	H	-4.9239	-0.24691	-1.2786	H	-4.88533	-0.6078	-1.32475	H	-4.7525	-0.58997	-1.56988
H	-1.49177	-0.83165	1.362686	H	-1.40135	-0.43332	1.526348	H	-1.34622	-0.67046	1.47704	H	-1.25652	-0.99162	1.25828
H	-4.88618	-3.33598	0.854945	H	-5.21364	-2.34875	1.887039	H	-4.91081	-3.01492	1.631162	H	-4.63399	-3.6576	0.734435
H	-3.70938	-2.97491	2.153183	H	-3.85178	-1.92988	2.969682	H	-3.5968	-2.55403	2.754806	H	-3.40857	-3.34915	2.00136
H	-5.19331	-2.00143	2.003054	H	-5.17789	-0.76811	2.718943	H	-5.04484	-1.52714	2.611385	H	-4.94933	-2.45658	2.018864
H	-0.07761	-0.0837	2.925289	H	0.160211	0.344718	2.90906	H	0.144116	0.144694	2.909554	H	0.293744	-0.38164	2.780942
H	-0.43745	-1.30496	4.144603	H	0.641198	-0.7706	4.196662	H	0.714481	-1.02905	4.104826	H	-0.00191	-1.66399	3.955396
H	1.78382	-0.5002	4.604445	H	2.539429	-1.58968	2.802302	H	2.707345	-1.5194	2.691095	H	2.288493	-0.95225	4.242387
H	1.928263	-2.14599	3.986343	H	2.073847	-0.51209	1.492722	H	2.166278	-0.36215	1.481756	H	2.302002	-2.57154	3.541405
H	2.59003	-1.28645	1.750327	H	2.440564	1.47622	3.017893	H	2.24803	1.469747	3.239605	H	2.687308	-1.5942	1.267396
H	2.324361	0.371368	2.270803	H	2.99639	0.38346	4.278707	H	2.953096	0.319122	4.367342	H	2.696921	0.025866	1.949323
H	4.141832	0.132873	3.982514	H	4.847978	-0.40462	2.747612	H	4.188631	1.192565	1.690565	H	4.697836	-2.19712	2.636784
H	4.413606	-1.52551	3.470989	H	4.298957	0.722445	1.516515	H	4.642441	1.799149	3.274169	H	4.973901	-0.90569	1.477983

H	5.087453	-0.72759	1.177032	H	4.769825	2.660431	3.06934	H	5.403377	-0.52907	3.943302	H	4.77153	0.806999	3.328315
H	4.779241	0.942838	1.652127	H	5.330241	1.527154	4.308568	H	5.023468	-1.10306	2.311626	H	4.562898	-0.49802	4.50726
H	6.088437	0.032179	2.426093	H	6.221575	1.68983	2.785938	H	6.288141	0.106191	2.546366	H	6.083456	-0.34405	3.62191

Conf. E				Conf. F				Conf. G			
C	-3.22988	4.296496	-0.4481	C	-4.61265	2.967111	1.351922	C	-2.16623	4.791748	0.333216
C	-4.21357	3.316027	-0.24612	C	-5.20393	1.741969	1.006845	C	-2.97131	4.130527	-0.6071
C	-3.92239	1.957454	-0.34195	C	-4.48751	0.74298	0.352201	C	-2.55967	2.949645	-1.22014
C	-2.61508	1.574205	-0.66876	C	-3.14939	0.988722	0.017456	C	-1.29943	2.430991	-0.89539
C	-1.6069	2.5605	-0.84146	C	-2.53567	2.214379	0.391836	C	-0.49273	3.077641	0.079227
C	-1.9229	3.922066	-0.73393	C	-3.2756	3.202171	1.056658	C	-0.93173	4.262047	0.687628
N	-2.03384	0.294973	-0.82462	N	-2.18973	0.175283	-0.62821	N	-0.63171	1.267888	-1.34347
C	-0.66502	0.482701	-1.07124	C	-0.97815	0.883985	-0.63746	C	0.57776	1.180551	-0.63571
C	-0.36603	1.867094	-1.08132	C	-1.1534	2.142104	-0.01154	C	0.692251	2.275224	0.255358
C	0.37759	-0.44517	-1.22161	C	0.299841	0.50987	-1.07981	C	1.571187	0.189646	-0.63751
C	1.720901	-0.01651	-1.38814	C	1.413389	1.375518	-0.91911	C	2.687712	0.270211	0.235697
C	1.989414	1.368515	-1.40932	C	1.208428	2.631071	-0.30895	C	2.786446	1.379118	1.102122
C	0.960711	2.290176	-1.26001	C	-0.05445	3.003644	0.134481	C	1.804934	2.36244	1.107084
N	0.338567	-1.82856	-1.20536	N	0.719112	-0.67083	-1.6681	N	1.65334	-0.97674	-1.37904
C	1.62981	-2.31497	-1.37846	C	2.08539	-0.58604	-1.91455	C	2.815928	-1.65165	-1.02159
C	2.523363	-1.21578	-1.49152	C	2.55822	0.673452	-1.45666	C	3.486686	-0.90998	-0.0121
C	2.075817	-3.63874	-1.44573	C	2.937402	-1.52462	-2.5054	C	3.324726	-2.85764	-1.5135
C	3.439397	-3.84869	-1.63682	C	4.280745	-1.1804	-2.63694	C	4.527624	-3.31341	-0.97901
C	4.337184	-2.77221	-1.74993	C	4.765599	0.06049	-2.18683	C	5.202532	-2.59347	0.022707
C	3.893181	-1.45578	-1.67747	C	3.917547	0.992416	-1.59618	C	4.693695	-1.39518	0.513513
C	3.27672	2.10199	-1.56137	C	2.169023	3.728567	-0.00803	C	3.823781	1.727373	2.112289
N	2.940402	3.437747	-1.47668	N	1.41563	4.704909	0.610743	N	3.392844	2.911098	2.675432
C	1.521906	3.692515	-1.30646	C	0.009074	4.379278	0.756777	C	2.148538	3.420657	2.129621
O	4.411439	1.670396	-1.72553	O	3.370506	3.803905	-0.23498	O	4.850621	1.133855	2.419058
C	-2.74986	-0.99997	-1.04274	C	-2.45565	-1.02621	-1.47851	C	-0.92584	0.485452	-2.58412
C	-3.16712	-1.67615	0.297974	C	-2.55862	-2.33327	-0.63674	C	-2.01679	-0.60104	-2.34848
C	-1.99246	-2.28993	1.076896	C	-1.20954	-2.84044	-0.10117	C	-1.52543	-1.81035	-1.53499
C	-1.23866	-3.24747	0.139622	C	-0.2337	-2.96428	-1.28246	C	-0.26398	-2.36899	-2.21297
C	-0.87347	-2.63124	-1.21432	C	-0.16176	-1.71528	-2.16603	C	0.80157	-1.3098	-2.50998
O	-1.9268	-1.87849	-1.79977	O	-1.4335	-1.15386	-2.45894	O	0.271277	-0.11391	-3.06438
C	-3.96303	-0.80684	-1.96191	C	-3.7125	-0.82313	-2.33478	C	-1.31779	1.415805	-3.73959
O	-3.89413	-0.71856	1.04022	O	-3.51514	-2.10401	0.377437	O	-3.1348	0.046838	-1.77958

N	-1.15488	-1.2559	1.665255	N	-0.72354	-2.00701	0.988716	N	-1.34925	-1.47313	-0.13051
C	-4.61804	-1.23555	2.147481	C	-3.95816	-3.26713	1.061746	C	-4.32676	-0.72459	-1.74573
C	-0.25007	-1.55432	2.646938	C	0.233322	-2.47179	1.849651	C	-1.30047	-2.45143	0.823989
C	0.521186	-0.37158	3.220156	C	0.553463	-1.57031	3.038686	C	-1.1202	-1.96518	2.259546
C	2.040085	-0.60596	3.134472	C	2.06112	-1.50592	3.329087	C	-1.86541	-2.84581	3.274109
C	2.881632	0.464095	3.845421	C	2.855294	-0.76689	2.243583	C	-3.39881	-2.79942	3.172794
C	2.795856	1.869436	3.232349	C	4.354198	-0.61066	2.546253	C	-4.03045	-1.4374	3.492973
C	3.728168	2.87433	3.917316	C	5.135563	-1.92983	2.577638	C	-5.56237	-1.46773	3.458911
O	-0.06534	-2.70956	3.022403	O	0.784285	-3.55553	1.672166	O	-1.36374	-3.64227	0.527243
H	-3.48822	5.348215	-0.36425	H	-5.19758	3.725207	1.864808	H	-2.51661	5.710049	0.795648
H	-5.22695	3.617055	0.005511	H	-6.24259	1.556019	1.266626	H	-3.94728	4.537276	-0.85785
H	-4.68392	1.225199	-0.11768	H	-4.94941	-0.21356	0.156659	H	-3.2259	2.429316	-1.89259
H	-1.15314	4.676361	-0.86921	H	-2.80754	4.140204	1.340828	H	-0.31477	4.759215	1.430536
H	1.395165	-4.47882	-1.34387	H	2.578005	-2.49225	-2.84308	H	2.805111	-3.43039	-2.27609
H	3.814148	-4.86726	-1.69263	H	4.966018	-1.89053	-3.09205	H	4.946476	-4.24833	-1.34166
H	5.395038	-2.97349	-1.89345	H	5.820419	0.293638	-2.30155	H	6.135856	-2.98177	0.420646
H	4.57227	-0.61459	-1.76384	H	4.275674	1.954798	-1.24709	H	5.203495	-0.82779	1.284493
H	3.638868	4.155895	-1.59381	H	1.833564	5.575282	0.902311	H	3.941337	3.379545	3.380386
H	1.108428	4.275864	-2.14284	H	-0.63368	5.101375	0.231333	H	2.279883	4.411984	1.670615
H	1.316895	4.252311	-0.38177	H	-0.30033	4.371217	1.812734	H	1.372532	3.516918	2.903761
H	-3.82752	-2.51333	0.017362	H	-2.92709	-3.10641	-1.33128	H	-2.27548	-0.98566	-3.34927
H	-2.38807	-2.9023	1.896287	H	-1.34432	-3.85354	0.296495	H	-2.281	-2.60317	-1.58221
H	-1.88618	-4.11304	-0.05183	H	-0.57113	-3.7973	-1.91294	H	-0.56323	-2.81703	-3.16941
H	-0.34032	-3.61508	0.641175	H	0.760139	-3.22085	-0.90804	H	0.15804	-3.16698	-1.59697
H	-0.70203	-3.44183	-1.93101	H	0.227898	-2.00566	-3.14796	H	1.46405	-1.69089	-3.29504
H	-3.64831	-0.29407	-2.87411	H	-3.6248	0.115106	-2.88813	H	-0.55808	2.192919	-3.8541
H	-4.34273	-1.79582	-2.23167	H	-3.76994	-1.64635	-3.05169	H	-1.3478	0.820708	-4.65609
H	-4.76309	-0.24092	-1.49008	H	-4.62566	-0.80738	-1.7442	H	-2.2893	1.88226	-3.59238
H	-1.36874	-0.29171	1.454621	H	-1.23878	-1.1681	1.215479	H	-1.39442	-0.50209	0.143261
H	-5.28263	-2.05722	1.841302	H	-4.32458	-4.02917	0.357792	H	-5.122	-0.0481	-1.42459
H	-3.95453	-1.59102	2.945972	H	-3.16779	-3.70539	1.684093	H	-4.57321	-1.12366	-2.74106
H	-5.22196	-0.41072	2.532297	H	-4.78048	-2.95017	1.707097	H	-4.25913	-1.55593	-1.03292
H	0.237567	0.559586	2.716316	H	0.146147	-0.56311	2.881571	H	-0.04281	-2.00514	2.470526
H	0.229237	-0.27369	4.27492	H	0.030489	-1.99437	3.907267	H	-1.40982	-0.91115	2.350852
H	2.248221	-1.58928	3.570174	H	2.209669	-1.00716	4.296685	H	-1.53163	-3.87822	3.125924
H	2.336452	-0.66289	2.078058	H	2.425682	-2.53302	3.436439	H	-1.55621	-2.54921	4.285258
H	2.592263	0.511695	4.906416	H	2.738248	-1.29312	1.286485	H	-3.70298	-3.12125	2.166773

H	3.931633	0.139665	3.832078	H	2.419487	0.231762	2.09938	H	-3.81146	-3.54821	3.863622
H	3.041229	1.810912	2.162536	H	4.791958	0.044208	1.782012	H	-3.69383	-1.10605	4.4861
H	1.763482	2.241116	3.28968	H	4.478304	-0.087	3.505631	H	-3.67275	-0.67906	2.783099
H	3.490339	2.973672	4.983899	H	4.783936	-2.5978	3.372295	H	-5.96057	-2.18436	4.187721
H	4.775426	2.557489	3.84056	H	5.03533	-2.46649	1.626204	H	-5.98881	-0.48487	3.690328
H	3.647932	3.869371	3.464541	H	6.203472	-1.74976	2.748101	H	-5.93095	-1.7667	2.469602
